# Supplementary material for: Basal actomyosin pulses expand epithelium coordinating cell flattening and tissue elongation
Source: Nat Commun. 2024 Apr 8;15:3000. doi: 10.1038/s41467-024-47236-1 (PMC11001887; doi:10.1038/s41467-024-47236-1)
Supplement: Supplementary file 1 — Supplementary Information [file 41467_2024_47236_MOESM1_ESM.pdf]

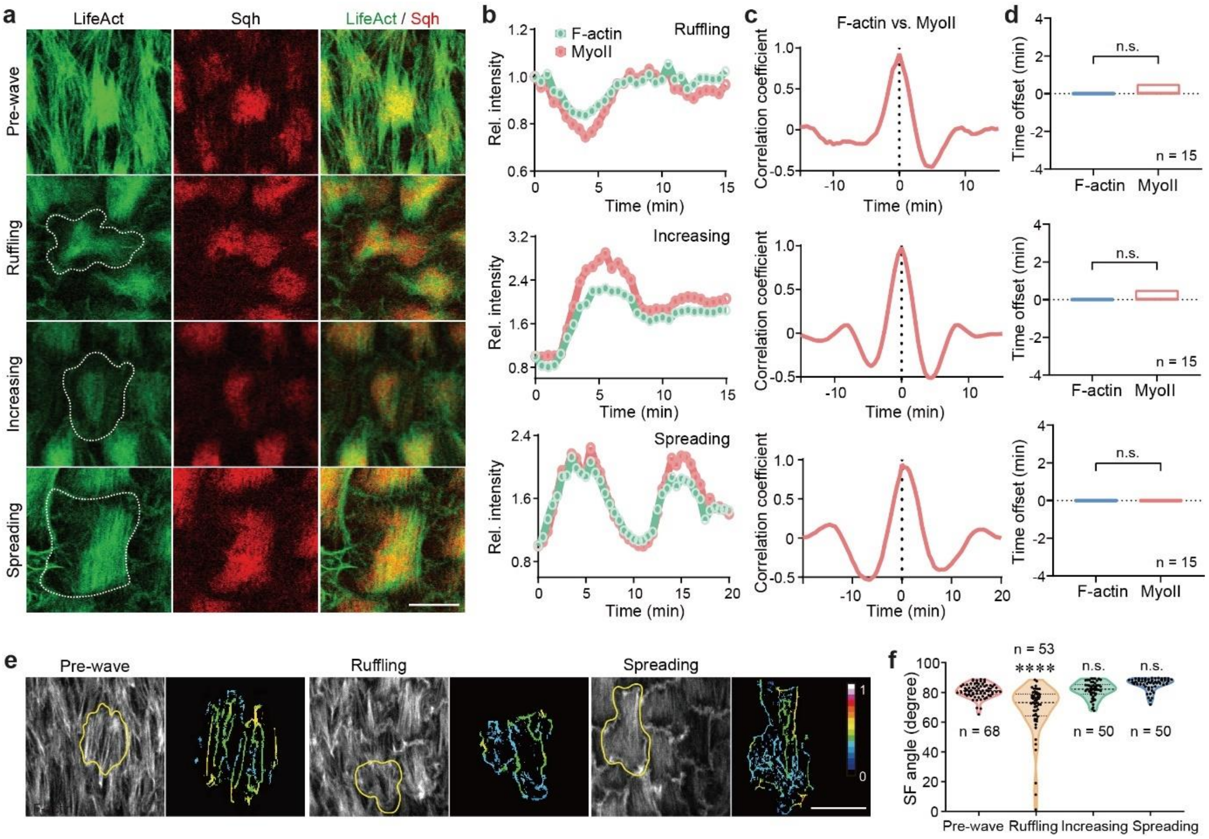

Supplementary Figure 1. Gradual modulation of basal actomyosin networks during follicle cell expansion behaviour.

a, Representative images of basal domains of follicle cells labelled with LifeAct-GFP and Sqh-RFP during the indicated phases. The experiments were repeated 20 times independently.

b, Quantifications of dynamic changes of basal F-actin and Myosin-II signals in one representative follicle cell during the indicated phases.

c, d, Quantifications of correlation coefficients (**c**) and time offsets (**d**) of basal F-actin and Myosin-II signals in follicle cells during the indicated phases. n = 15 independent follicle cells in (**d**).

e, Representative images and segmentations of basal stress fiber networks in posterior follicle cells during the indicated phases. Yellow circles mark one cell boundary. RGB bar means intensities of individual stress fiber networks.

f, Quantification of basal stress fiber A-P polarity during the indicated phases. n = 68, 53, 50, 50 independent follicle cells during the indicated phases.

Scale bars are 10 μm in (**a, e**). The middle line indicates the median, and floating bars shows the minimum to maximum, in (**d**). The middle line shows medians, upper and lower lines as 25th and 75th percentiles, each datapoint is displayed as a dot, in (**f**). The dashed lines in (**c**) indicate the time point of signal delay. All P values have been listed in Supplementary Note 1. Source data are provided as a Source Data file.

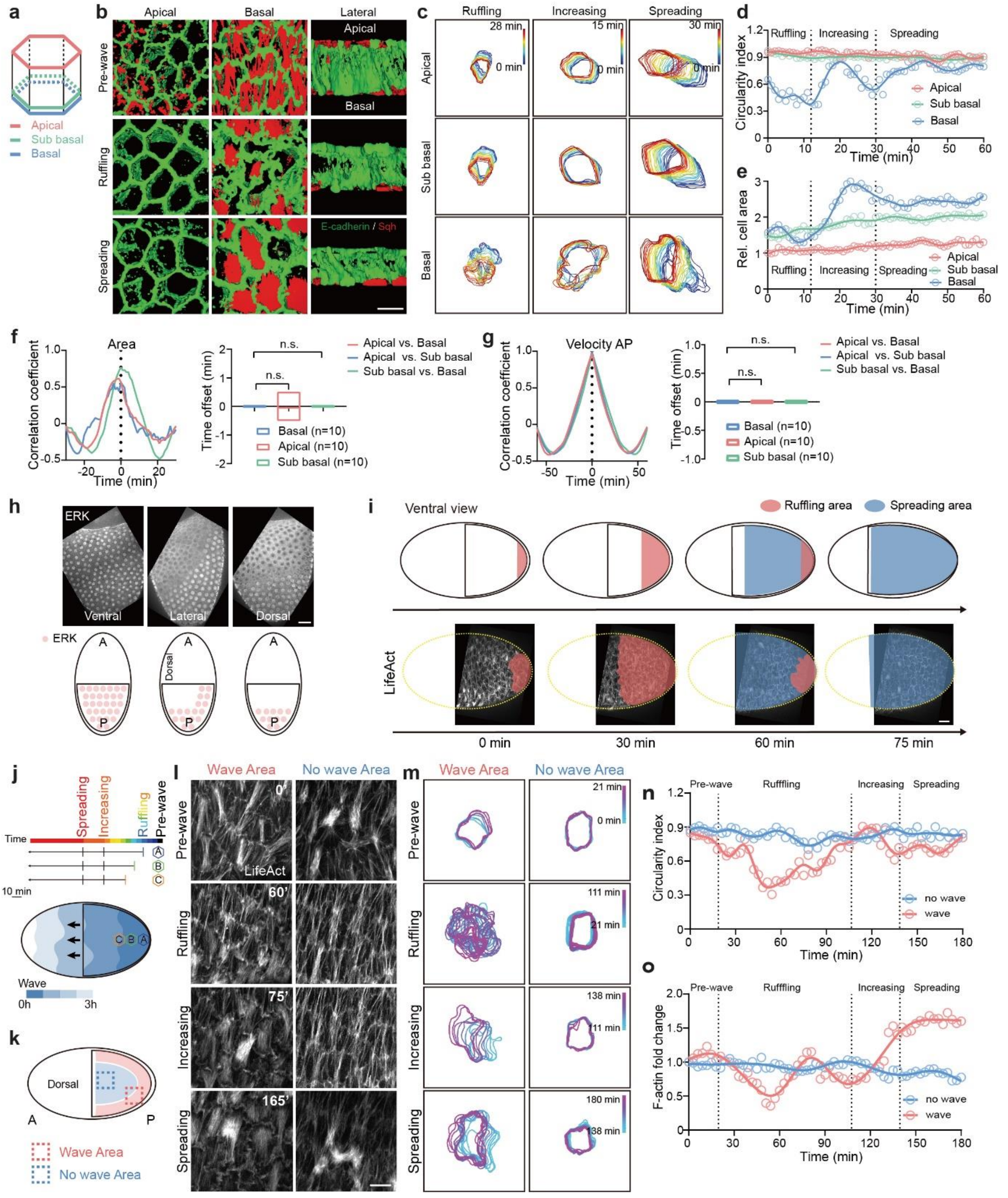

Supplementary Figure 2. Follicle cell expansion behaviours.

a, Representative cartoon to summarize different subcellular domains (labelled by red, green and blue colours).

b, Representative images of posterior follicular cells labelled with E-cadherin-GFP and Sqh-RFP at apical, basal and lateral views, during the indicated phases.

c, Boundary outlines mark the temporal changes of different subcellular domains.

d, e, Quantifications of dynamic changes of circularity index (**d**) and cell area (**e**) in different subcellular domains.

f, g, Quantifications of correlation coefficient (left) and time offset (right) of area change (**f**) and A-P migration velocity (**g**) between different subcellular domains of n = 10 indicated follicle cells.

h, Representative images (above) and cartoons (below) of follicle cells labelled with ERK-GFP reporter (pink colour) during the ruffling phase, at different views.
i, Representative time-lapse images of follicular cell basal domains monitored by LifeAct-RFP tracked at the tissue ventral view, showing cell expansion waves. Pink and blue colours label ruffling and spreading area, respectively.
j, Representative cartoon to summarize the P-to-A propagation of cell expansion waves. Different blue colors mean the most active wave region at different time points. A, B and C represent three positions in the tissue posterior region, and RGB colours mark different phase occurrence periods.
k, l, Representative cartoon (**k**) and images (**l**) of follicle cell basal domains monitored by LifeAct-RFP in different tissue regions.
m, Boundary outlines mark temporal changes (colour bars) of two different follicle cells.
n, o, Quantifications of dynamic changes of circularity index (**n**) and basal F-actin intensity (**o**) of two different representative follicle cells.
Scale bars are 10 μm in (**b, l**), and 20 μm in (**h, i**). The middle line indicates the median, and floating bars shows the minimum to maximum, in (**f, g**). The dashed lines in (**d**) indicate the starting time points of increasing and spreading processes. The dashed lines in (**f, g**) indicate the time points of signal delay. The dashed lines in (**n, o**) indicate the starting time points of ruffling, increasing and spreading processes. All P values have been listed in Supplementary Note 1. Source data are provided as a Source Data file.

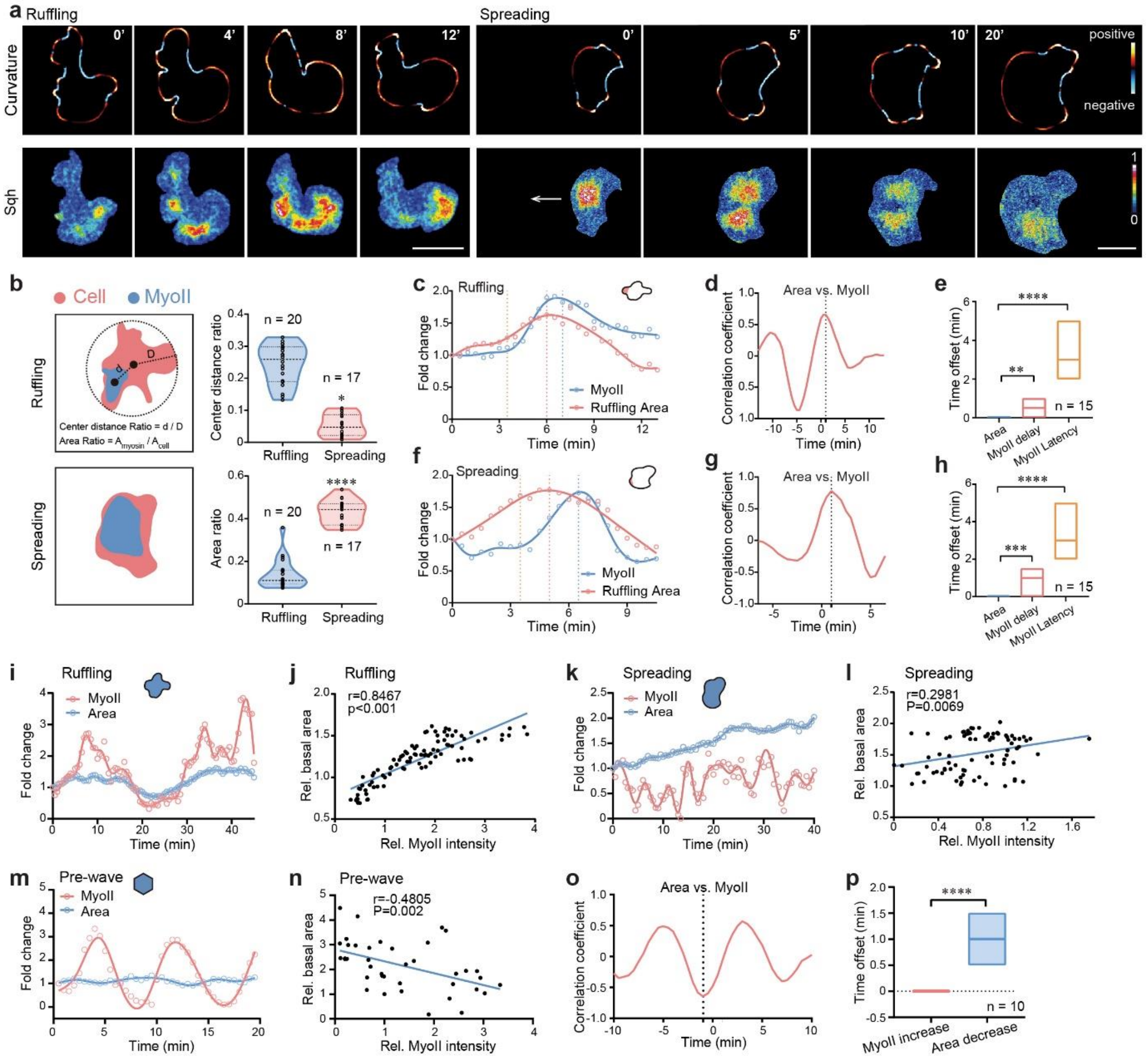

Supplementary Figure 3. Correlations between basal myosin pulses and surface area display distinct patterns between the two phases.

a, Representative time-lapse images of curvature values (reflected as colour bars) and basal Myosin signals (heatmap) in one follicle cell during the ruffling and spreading phases. Arrow marks migration direction.

b, Representative quantification cartoons and values for different subcellular localizations of basal actomyosin networks. Pink and blue colours mean cell and Myosin-II, respectively.

c-h, Quantifications of dynamic changes (**c, f**), correlation coefficients (**d, g**) and time off-sets (**e, h**) of local basal Myosin-II signals and local ruffle-associated area changes during ruffling (**c-e**) and spreading (**f-h**) phases.

i, k, Quantifications of dynamic changes of total basal surface area (blue cartoon) and basal Myosin-II signals during the ruffling (**i**) and spreading (**k**) phases.

j, l, Scatter plot comparing a random sample of points in follicle cells for total basal surface area and basal Myosin-II intensity during the ruffling (**j**) and spreading (**l**) phases.

m, Quantification of dynamic changes of total basal surface area (blue cartoon) and basal Myosin-II signals in one representative follicle cell during the pre-wave phase.

N, Scatter plot comparing a random sample of points in follicle cells for total basal surface area and basal Myosin-II intensity during the pre-wave phase.

o, p, Quantifications of the correlation coefficient of total basal Myosin-II signals and total basal area (**o**) and time off-set of total Myosin-II signals and area decrease (**p**) during the pre-wave phase.
Scale bars are 10 μm in (**a**). The middle line shows medians, upper and lower lines as 25th and 75th percentiles, each data-point is displayed as a dot in (**b**). The middle line indicates the median, and floating bars shows the minimum to maximum, in (**e, h, p**). The dashed lines in (**c, f**) indicate the peaks of corresponding colour-marked signals within one cycle, while the yellow dashed lines in (**c, f**) indicate the end time of Myosin-II signal latency. The dashed lines in (**d, g, o**) indicate the time points of signal delay. All P values have been listed in Supplementary Note 1. Source data are provided as a Source Data file.

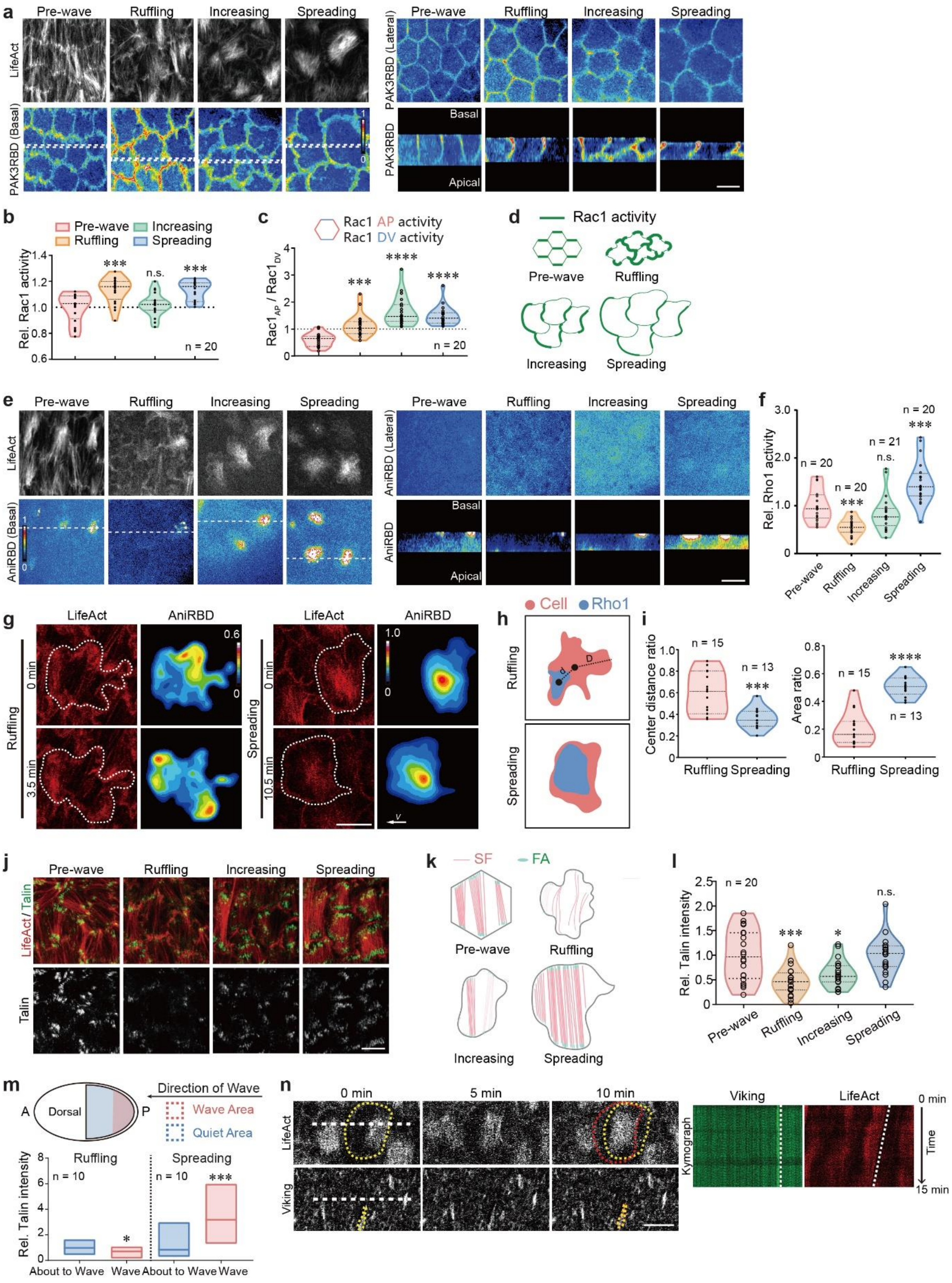

Supplementary Figure 4. Rac1 and Rho1 activities as well as focal adhesions during follicle cell expansion behaviour.

a, e, j, Representative PAK3RBD-GFP (heatmap) and LifeAct-RFP images (**a**), AniRBD-GFP (heatmap) and LifeAct-RFP images (**e**) in follicle cells from basal view, lateral view, and apical-to-basal view, as well as Talin-GFP and LifeAct-RFP images (**j**), during the indicated

phases. Dotted lines show the region where the section from apical-to-basal view has been chosen in (**a, e**).
b, c, f, l, Quantifications of basal Rac1 activity (**b**) and Rac1 activity polarity (Rac1 A-P/Rac1 D-V) (**c**), basal Rho1 activity (**f**), and total Talin intensity (**l**) in follicle cells.
d, h, k, Representative cartoon to summarize basal Rac1 activity polarization (green label, **d**), subcellular localizations of basal Rho1 activity (blue label versus pink labelled cell, **h**), and focal adhesions (green label) and basal stress fibers (red label) (**k**).
g, Representative time-lapse images of basal AniRBD-GFP (heatmap) and LifeAct-RFP images in one follicle cell during the ruffling and spreading phases. Dotted lines show membrane boundary of one representative follicle cell at different time points.
i, Quantifications for different subcellular localizations of basal Rho1 activity during the two indicated phases.
m, Quantification of total Talin intensity in follicle cells within wave-occurring vs. other regions during the ruffling and spreading phases.
n, Representative time-lapse images and kymograph of Collagen VI (Viking-GFP) and basal stress fibers (LifeAct-RFP) in follicle cells during the spreading phase. Dotted circles mark the boundary of either one follicle cell or one matrix fiber. Dotted lines mark the lines used for kymograph analysis of Collagen VI and stress fibers. The experiments were repeated 10 times independently.
Scale bars are 10 μm in (**a, e, g, j, n**). The middle line shows medians, upper and lower lines as 25th and 75th percentiles, each data-point is displayed as a dot, in (**b, c, f, i, l**). The middle line indicates the median, and floating bars shows the minimum to maximum, in (**m**). RGB bars mean signal intensities in (**a, e, g**). All P values have been listed in Supplementary Note 1. Source data are provided as a Source Data file.

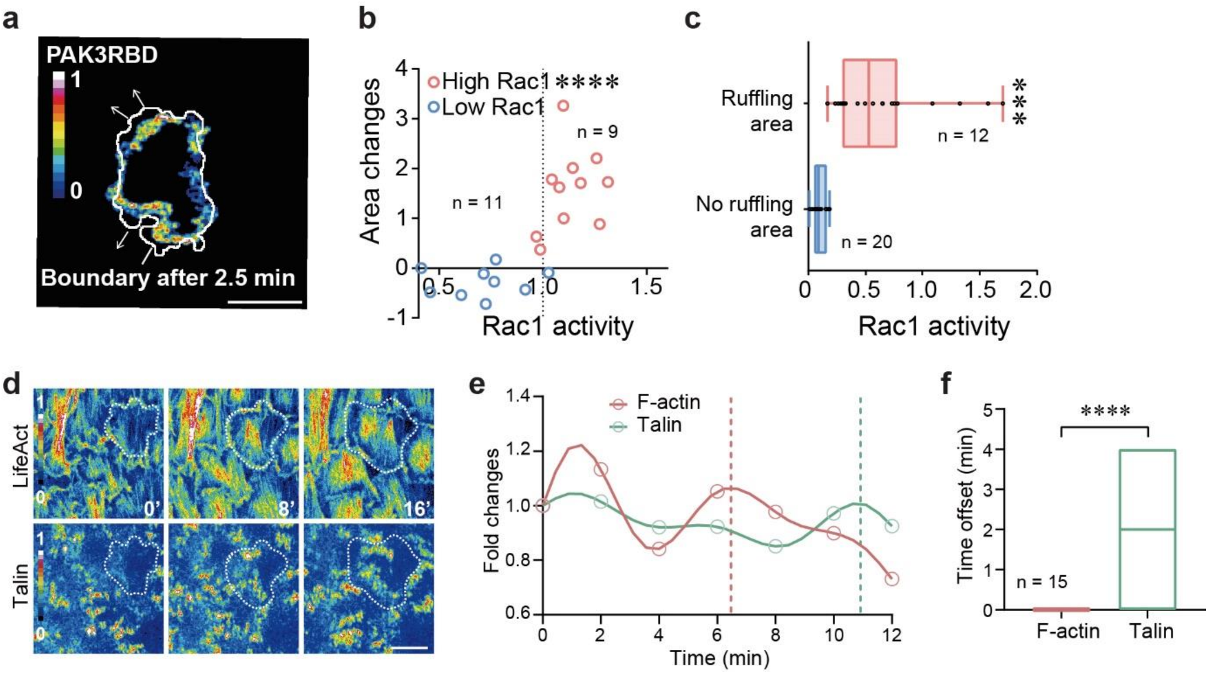

Supplementary Figure 5. Correlations between the Rac1 activity and membrane protrusions during cell ruffling behaviours as well as correlations between focal adhesions and stress fibers during the increasing phase.

a, The representative image showing local basal Rac1 activity followed by ruffling-associated membrane outward growth. White line marks the membrane boundary after 2.5 minutes of Rac1 activity, and arrows mark local membrane outward growth. RGB bar means signal intensities.

b, c, Quantifications of randomly (**b**) or anteriorly (**c**) distributed local Rac1 activity whose strength is correlated to ruffling-associated membrane outward growth, during the ruffling (**b**) and spreading (**c**) phases.

d, Representative time-lapse images of focal adhesions (monitored by Talin-GFP, shown as heat map) and basal stress fibers (monitored by LifeAct-RFP, shown as heat map) in follicle cells during the increasing phase. White dotted lines mark the whole cell boundary. RGB bars mean signal intensities.

e, f, Quantifications of dynamic change of Talin and stress fiber F-actin signals (**e**) and the time offset (**f**) in follicle cells during the increasing phase. The dashed lines in (**e**) indicate the peaks of corresponding colour-marked signals within one cycle. n = 15 independent follicle cells in (**f**).

Scale bars are 10 μm in (**a, d**). Boxplot shows medians, 25th and 75th percentiles as box limits, minimum and maximum values as whiskers; each datapoint is displayed as a dot in (**c**). The middle line indicates the median, and floating bars shows the minimum to maximum, in (**f**). All P values have been listed in Supplementary Note 1. Source data are provided as a Source Data file.

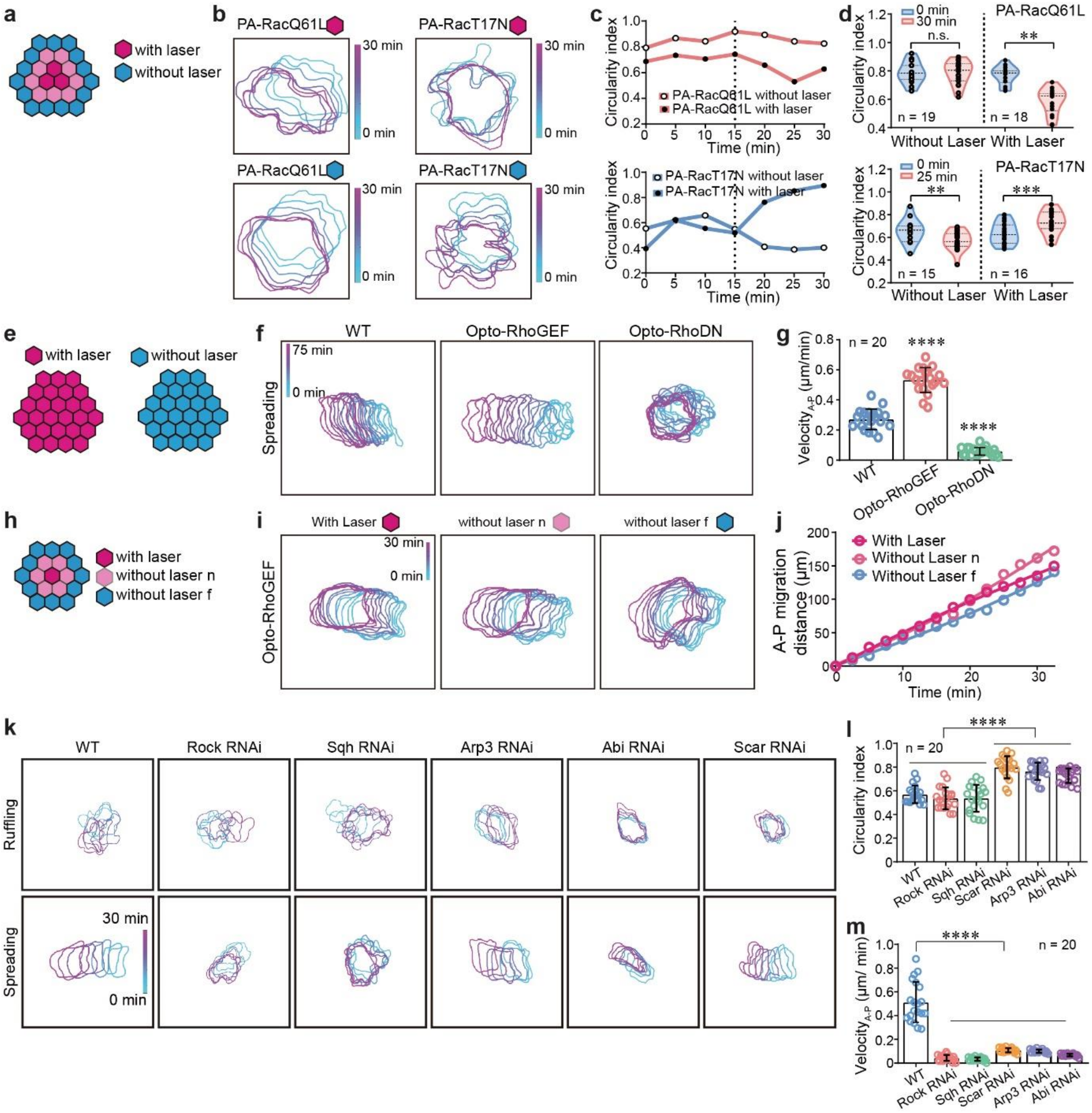

Supplementary Figure 6. The roles of Rac1 and Rho1 signaling in expanding follicle cells.
a, e, h, Representative cartoons to summarize various follicle cell states in optogenetic experiments of PA-Rac (**a**), global Opto-RhoGEF or Opto-RhoDN (**e**) and local Opto-RhoGEF (**h**). Dark pink hexagons mean photo-treated cells, blue hexagons mean either not photo-treated cells or cells far from photo-treatment, and light pink hexagons mean cells close to photo-treatment.
b, f, i, Boundary outlines mark the temporal changes (colour bars) of one representative follicle cell in optogenetic experiments of PA-Rac (**b**), global Opto-RhoGEF or Opto-RhoDN (**f**) and local Opto-RhoGEF (**i**).
c, Quantifications of dynamic changes of circularity index in one representative follicle cell with or without PA-Rac treatment. Dotted lines mark the beginning time of ruffling behaviour changes after Rac1 activity is enhanced or inhibited.
d, Quantifications of circularity index of follicle cells with or without PA-Rac treatment.
g, Quantification of migration velocity of n = 20 follicle cells with the indicated optogenetic conditions.
j, Dynamic quantification of migration distance of one representative follicle cell with the indicated optogenetic conditions. The experiments were repeated 10 times independently.

k, Boundary outlines mark the temporal changes (colour bars) of one representative indicated follicle cell during the ruffling (above) and spreading (below) phases.
l, m, Quantifications of circularity index during the ruffling phase (**l**), and migration velocity during the spreading phase (**m**) in the n= 20 indicated follicle cells.
The middle line shows medians, upper and lower lines as 25th and 75th percentiles, each datapoint is displayed as a dot, in (**d**). Box shows means and whiskers show SD; each datapoint is displayed as a dot in (**g, l, m**). All P values have been listed in Supplementary Note 1. Source data are provided as a Source Data file.

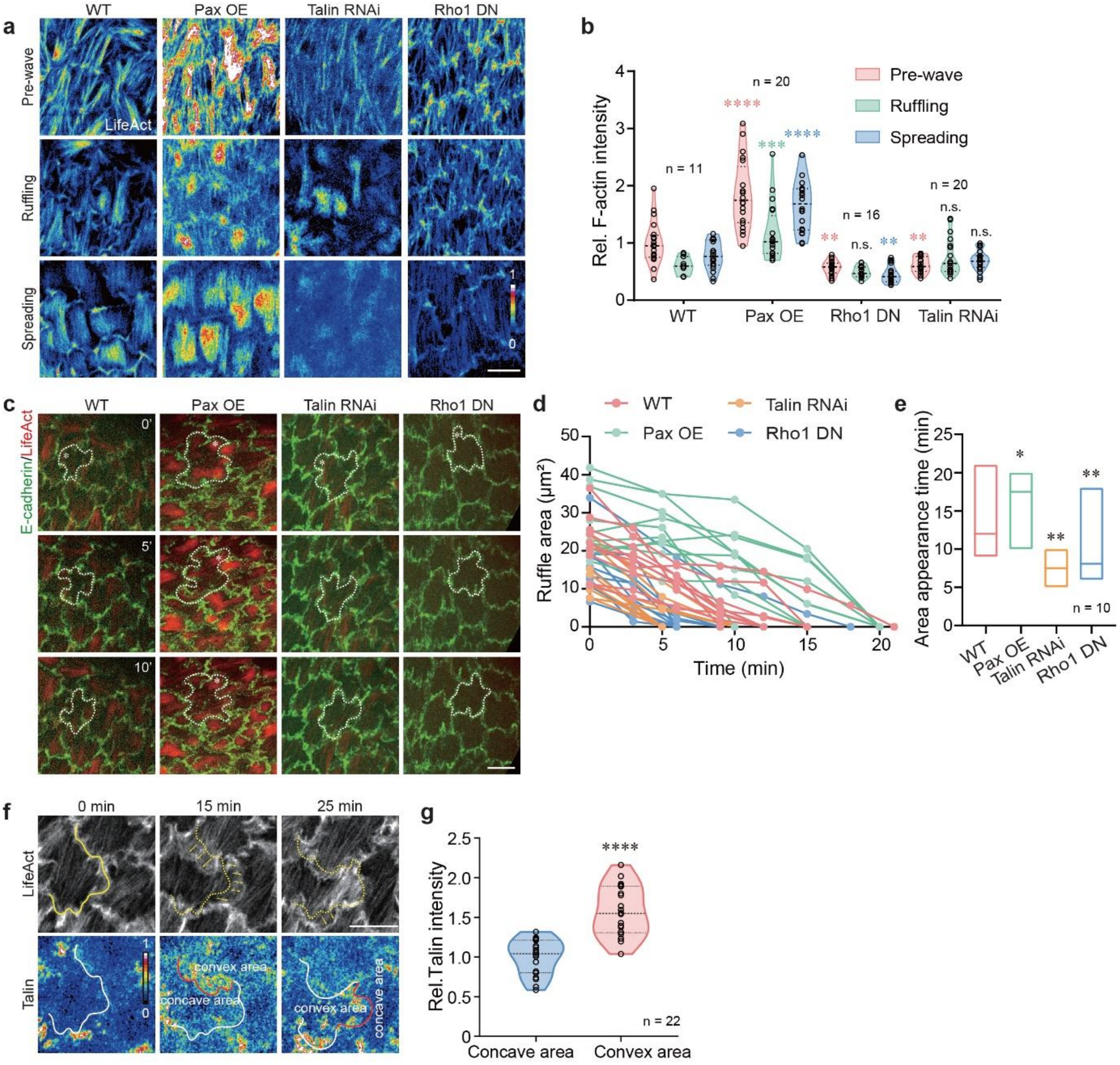

Supplementary Figure 7. Dynamics and roles of focal adhesions during follicle cell expansion behaviour.

a, b, Representative images (**a**, LifeAct-RFP shown in heatmap) and quantification (**b**) of basal stress fiber F-actin signals in follicle cells with the indicated genotypes during the indicated phases. n = 11, 20, 16, 20 indicated follicle cells in (**b**).

c-e, Representative E-cadherin-GFP and LifeAct-RFP images of ruffling status (**c**), dynamic quantification of ruffles-associated area decrease after area reaching the maximum (**d**), and the appearance time of ruffling-associated area (**e**) in follicle cells with the indicated genotypes during the ruffling phase. White dotted lines mark a whole membrane boundary region, and stars mark a local ruffling region. n = 10 independent follicle cells in (**e**).

f, g, Representative time-lapse images of Talin-GFP (shown in heatmap) and LifeAct-RFP in follicle cell basal domains (**f**), and quantification of focal adhesions at the concave (area decrease) and convex (area increase) regions (**g**) during the ruffling phase. In (**f**) upper panels, yellow dotted lines mark the cell boundary at one previous time point and yellow arrows marks membrane expansion direction; in (**f**) lower panels, white lines mark the cell boundary and red lines mark the boundary where concave and convex are concentrated. n = 22 independent follicle cells in (**g**).

Scale bars are 10 μm in (**a, c, f**). The middle line indicates the median, and floating bars shows the minimum to maximum, in (**e**). The middle line shows medians, upper and lower lines as 25th and 75th percentiles, each datapoint is displayed as a dot, in (**b, g**). RGB bars mean signal intensities in (**a, f**). All P values have been listed in Supplementary Note 1. Source data are provided as a Source Data file.

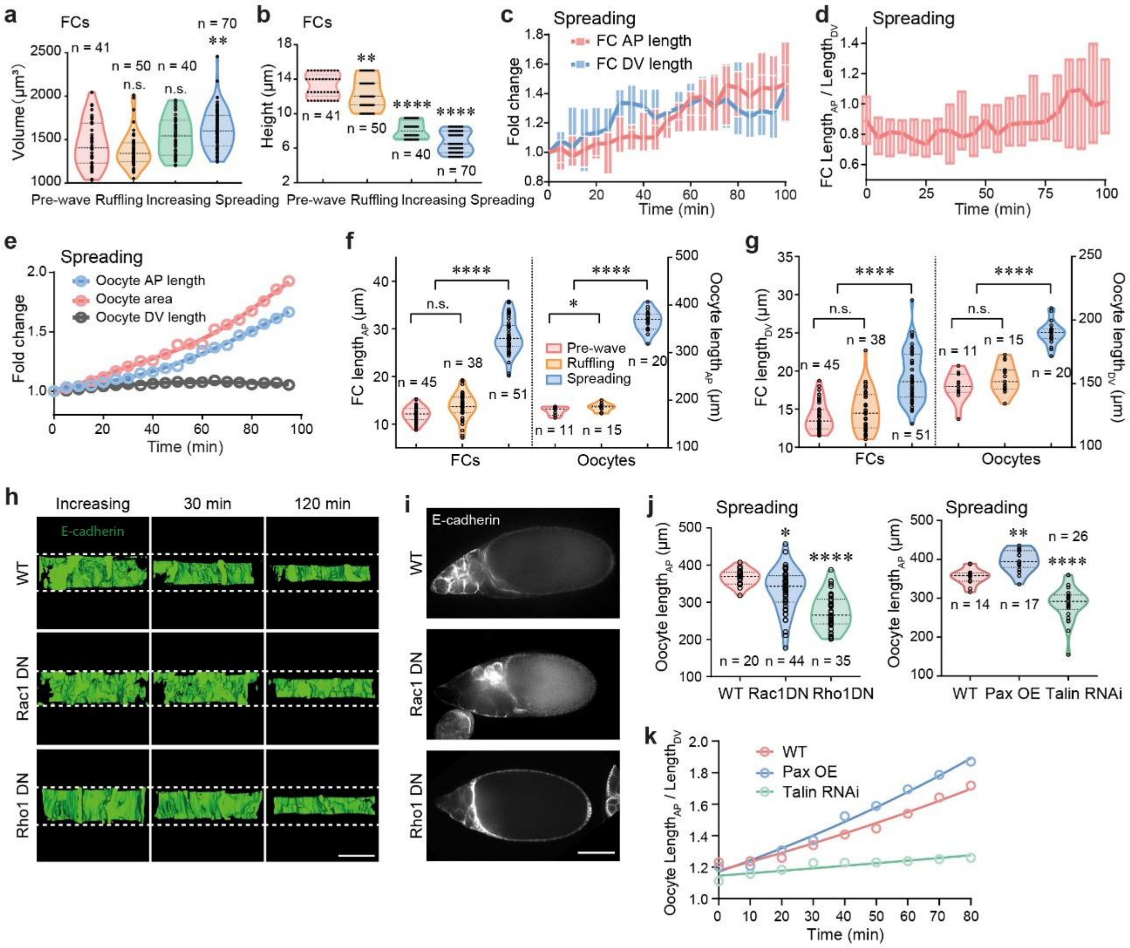

Supplementary Figure 8. Follicle cell expansion behaviour bridges epithelial cell flattening and elongation to oocyte growth and extension.

a, b, Quantifications of total cell volume (**a**) and cell height (**b**) of follicle cells during the indicated phases.

c, d, Quantifications of the dynamic changes of A-P length and D-V length (**c**) and A-P length/D-V length ratio (**d**) of follicle cells during the spreading phase.

e, Quantifications of the dynamic changes of A-P length, D-V length and area of one representative oocyte during the spreading phase.

f, g, Quantifications of A-P length (**f**) and D-V length (**g**) of follicle cells and oocytes during the indicated phases.

h, Representative time-lapse lateral view images of WT, Rac1DN-expressing, and Rho1-expressing follicle cells labelled with E-cadherin-GFP, showing their flattening state. The experiments were repeated 10 times independently.

i, Representative E-cadherin-GFP images of one egg chamber from WT, Rac1DN-expresssing or Rho1DN-expressing follicular epithelium after follicle cell expansion waves (during S12).

j, Quantifications of A-P length of the oocytes from the indicated follicular epithelia during the spreading phase.

k, Quantification of dynamic changes of A-P length/D-V length ratio of one representative oocyte from the indicated follicular epithelium during the spreading phase. The experiments were repeated 10 times independently.

Scale bars are 10 μm in (**h**), and 100 μm in (**i**). The middle line shows medians, upper and lower lines as 25th and 75th percentiles, each datapoint is displayed as a dot, in (**a, b, f, g, j**). Boxes show min to max, in (**c, d**). All P values have been listed in Supplementary Note 1. Source data are provided as a Source Data file.

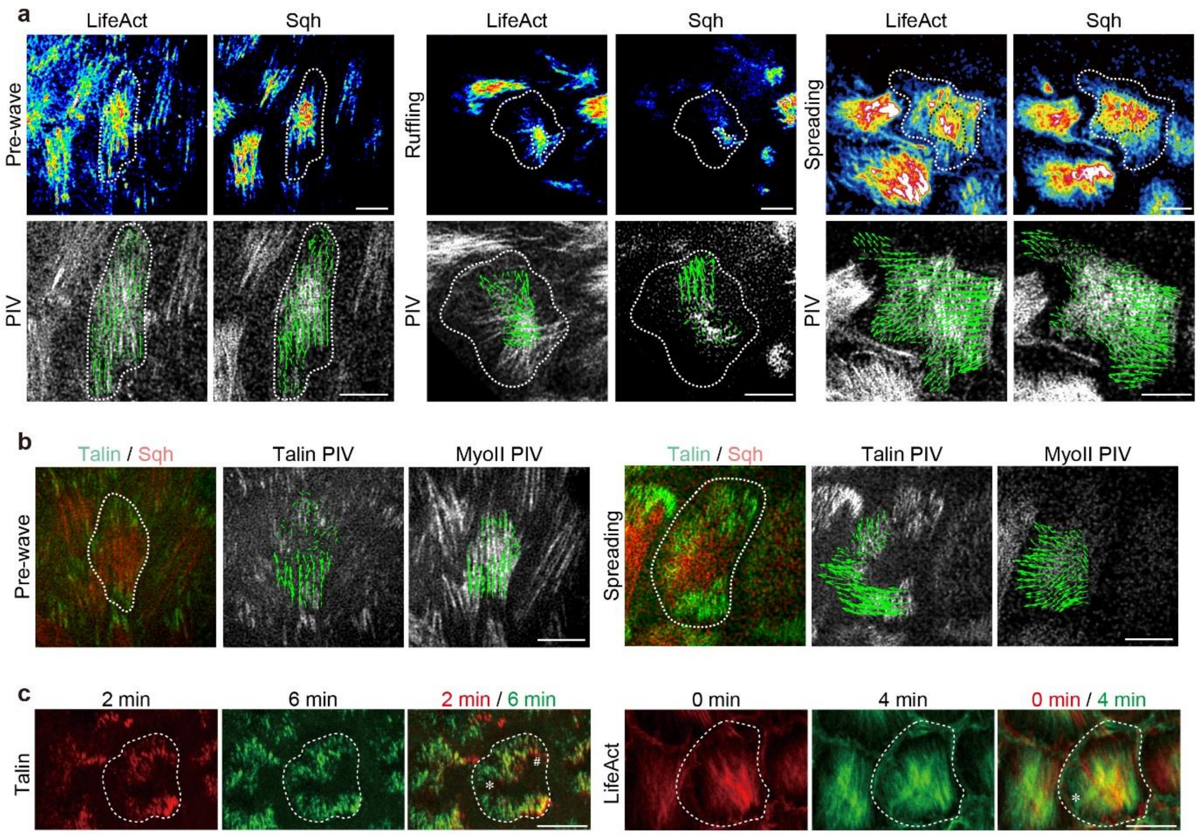

Supplementary Figure 9. Nanoscale dynamics of basal actomyosin networks and focal adhesions in follicle cells during different phases.

a, Nanoscale fiber dynamics of basal F-actin and Myosin-II signals in follicle cells during the pre-wave, ruffling and spreading phases, including original images of LifeAct-GFP and Sqh-RFP signals (above, shown as heatmap) and PIV processing data (below). White dotted circles mark the whole cell region of stress fibers at different phases, small white dotted circles mark the strong F-actin and Myosin-II signal regions in ruffling phase, and small black dotted circles mark the strong F-actin and Myosin-II signal regions in spreading phase.

b, Nanoscale fiber dynamics of basal Talin and Myosin-II signals in follicle cells during the pre-wave and spreading phases, including original Talin-GFP/Sqh-RFP images and their PIV processing data. White dotted circles mark the whole cell region of focal adhesions and stress fibers.

c, Representative time-lapse images of Talin-GFP and LifeAct-RFP showing how the D-V oriented actomyosin and focal adhesions assemble and disassemble at the anterior or posterior terminal of follicle cells, respectively, allowing cell movement in the P-to-A direction. Asterisk marks new assembly of focal adhesions and stress fibers at cell anterior edge, and hash marks gradual disassembly of focal adhesions at cell posterior edge. White dotted circles mark the whole cell region of focal adhesions and stress fibers. To analyze signal dynamics in the same region within a cell, we corrected the position of the cell in the images so that the cell did not appear displaced in the images at different time points.

Scale bars are 5 μm in (**a, b**) and 10 μm in (**c**).

The experiments in (**a-c**) were repeated 10 times independently.

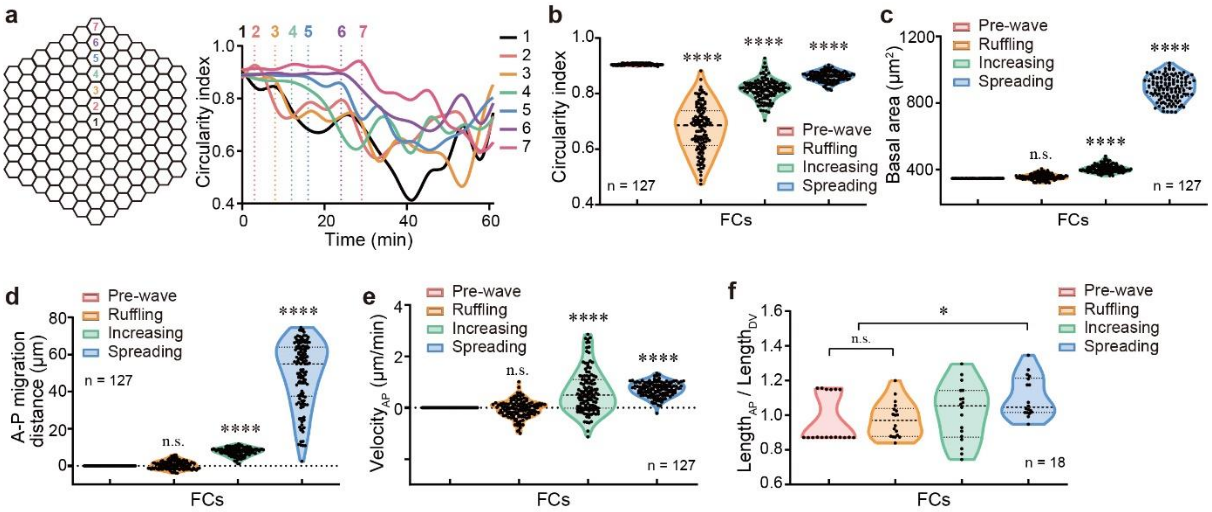

Supplementary Figure 10. The *in-silico* simulation of follicle cell expansion behaviour in WT tissues.

a, Representative cartoon to summarize follicle cells located at different positions (labelled by different colored numbers) from tissue posterior region (left). The simulation quantification of dynamic changes of circularity index during ruffling phase in follicle cells located at different positions (labelled by different colored numbers), confirming that waves initiate from tissue posterior region and propagate toward tissue anterior region. The dashed lines in (**a**) indicate the starting time points of ruffling behaviours in follicle cells at tissue different posterior positions. These simulated results were repeated 3 times independently.

b-f, Simulation quantifications of circularity index (**b**), basal surface area (**c**), migration distance (**d**), velocity (**e**) and A-P length/D-V length ratio (**f**) of WT follicle cells during the indicated phases. n = 127, 127, 127, 127, 18 independent simulated results in (**d-f**), respectively. The middle line shows medians, upper and lower lines as 25th and 75th percentiles, each data-point is displayed as a dot, in (**b-f**). All P values have been listed in Supplementary Note 1. Source data are provided as a Source Data file.

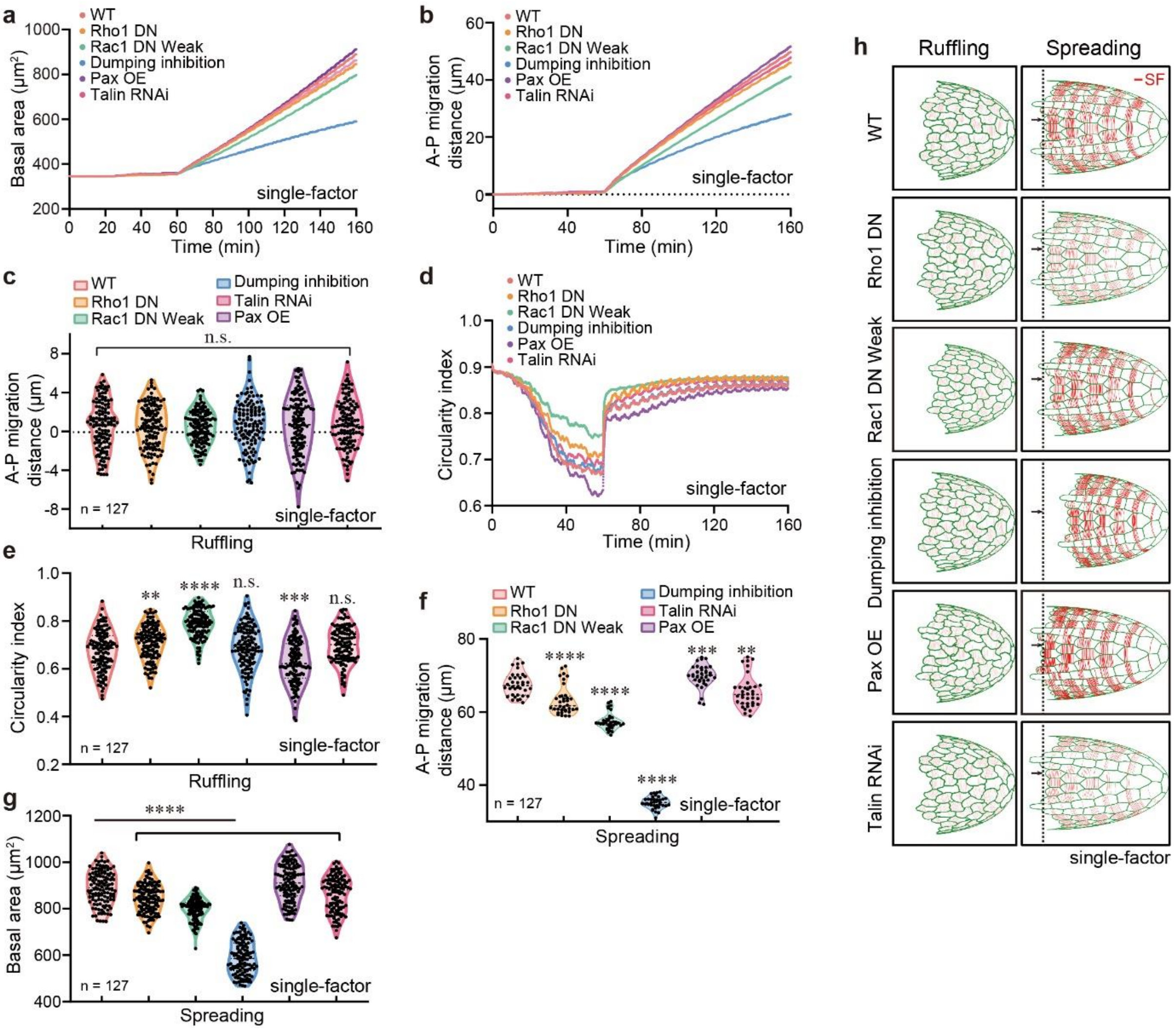

Supplementary Figure 11. Single-factor simulations of follicle cell expansion behaviour and oocyte elongation in tissues with different genetic backgrounds.

a, b, Single-factor simulation quantifications of dynamic changes of basal surface area (**a**) and migration distance (**b**) of follicle cells with the indicated genetic backgrounds, during whole cell expansion wave period. These simulated results in (**a, b**) were repeated 3 times independently.

c, Single-factor simulation quantification of migration distance of n = 127 follicle cells with the indicated genetic backgrounds during the ruffling phase.

d, Single-factor simulation of dynamic changes of circularity index of follicle cells with the indicated genetic backgrounds during whole expansion wave period. These simulated results were repeated 3 times independently.

e-g, Single-factor simulation quantifications of circularity index (**e**) during the ruffling phase, migration distance (**f**) and basal surface area (**g**) during the spreading phase in n = 127 follicle cells with the indicated genetic backgrounds.

h, Single-factor simulation of oocyte elongation and follicle cell spreading behaviour with the indicated genetic backgrounds during the ruffling and spreading phases. Dotted lines and arrows mark the final position of leading follicle cells in WT tissues. Green colours label cell membranes and red colours label basal stress fibers, in this simulation.

The middle line shows medians, upper and lower lines as 25th and 75th percentiles, each data-point is displayed as a dot, in (**c, e, f, g**). All P values have been listed in Supplementary Note 1. Source data are provided as a Source Data file.

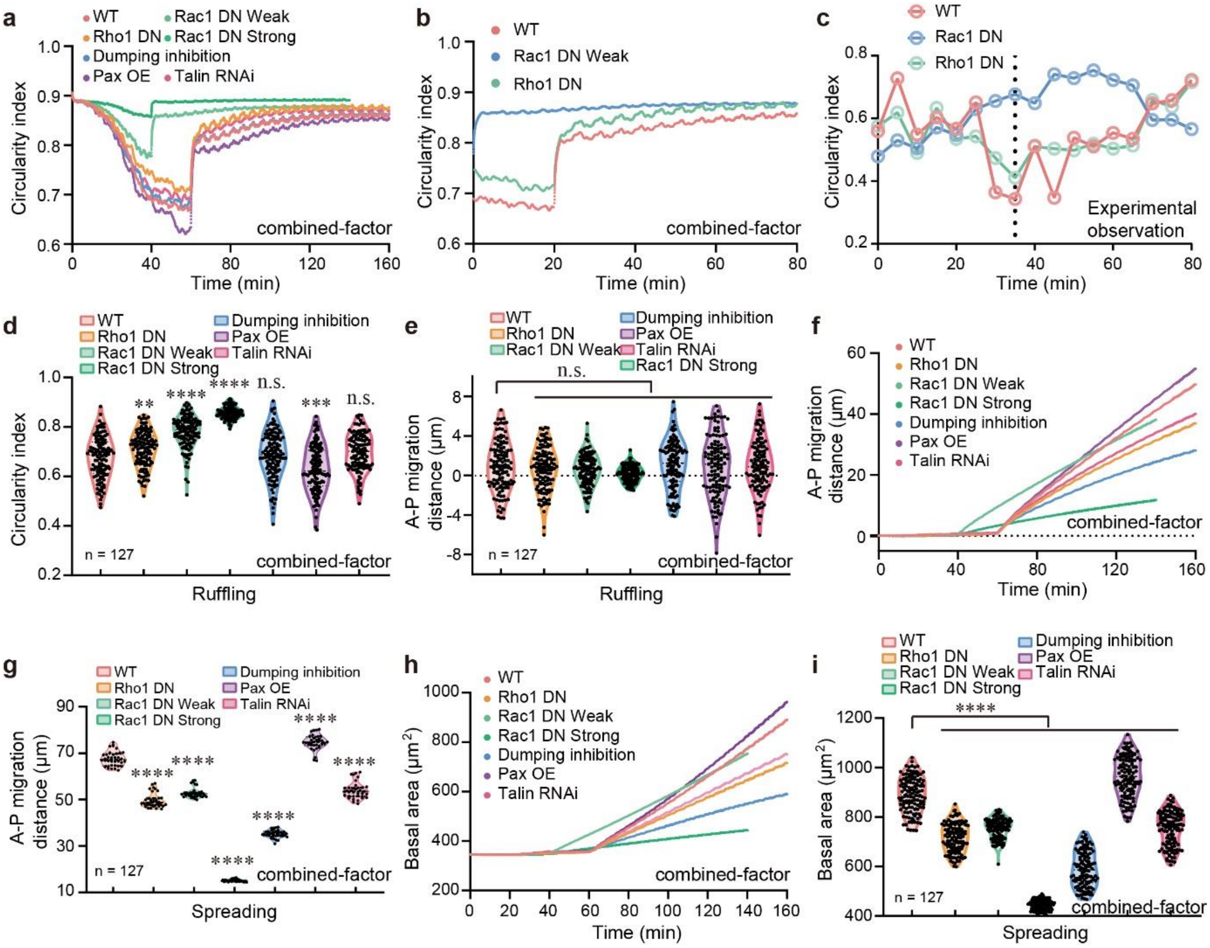

Supplementary Figure 12. Combined-factor simulations of follicle cell expansion behaviour in tissues with different genetic backgrounds.

To further explore the passive drive of follicle cell expansion, the effect of oocyte A-P extension was considered in the combined-factor simulation.

a, Combined-factor simulation quantification of dynamic changes of circularity index of follicle cells with the indicated genetic background, during whole cell expansion wave period. These simulated results were repeated 3 times independently.

b, c, Combined-factor simulation and experimental quantification of dynamic circularity index of follicle cells with the indicated genetic backgrounds during whole expansion wave period. Simulation in (**b**) includes late ruffling, increasing and spreading phases with a total time of 80 minutes, which is an excerpt from 40-120 minutes in (**a**). These simulated results and experimentally observed results in (**b, c**) were repeated 3 times independently.

d, e, Combined-factor simulation quantifications of circularity index (**d**) and migration distance (**e**) of follicle cells with the n = 127 indicated genetic backgrounds during the ruffling phase.

f, h, Combined-factor simulations of dynamic changes of migration distance (**f**) and basal surface area (**h**) of follicle cells with the indicated genetic backgrounds, during whole cell expansion wave period. These simulated results in (**f, h**) were repeated 3 times independently.

g, i, Combined-factor simulation quantifications of migration distance (**g**) and basal surface area (**i**) of follicle cells with the n = 127 indicated genetic backgrounds during the spreading phase.

The middle line shows medians, upper and lower lines as 25th and 75th percentiles, each data-point is displayed as a dot, in (**d, e, g, i**). The dashed line in (**c**) indicates the starting time point of ruffling behaviour. All P values have been listed in Supplementary Note 1. Source data are provided as a Source Data file.

Supplementary Table 1

Drosophila stock	*Reference or Source*
Sqh::RLCmyosinII–mCherry	(1)
Slbo::LifeAct–GFP	(2)
Slbo::LifeAct–RFP	(2)
E-cadherin-GFP	(3)
Talin-GFP	(4)
Ubi::AniRBD-GFP	(5)
Sqh::PAK3-RBD-GFP	made using BDSC (52303) (52304)
Singed^{2}/FM7	from Jennifer Zanet
Tj-Gal4	Kyoto Stock Center (#104055)
Nanos-Gal4	BDSC (#7017)
tubP-GAL80^{ts}	BDSC (#4937)
UASt-PA-RacCA	(2)
UASt-PA-RacDN	(2)
UASp-CIBN-CAAX	(6)
UASp-Cry2-RhoGEF	(6)
UASp-Cry2-Rho1DN	(7)
UAS-Paxillin	(8)
UAS-Rac1DN	BDSC (#6292)
UAS-Rho1DN	BDSC (#7327)
UAS-Rock^{dsRNA}	BDSC (#34324)
UAS-Sqh^{dsRNA}	BDSC (#32439)
UAS-Scar^{dsRNA}	BDSC (#51803)
UAS-Abi^{dsRNA}	BDSC (#51455)
UAS-Arp3^{dsRNA}	BDSC (#32921)
UAS-Talin^{dsRNA}	BDSC (#33913)
UAS-Singed^{dsRNA}	BDSC (#42615)

Supplementary Note 1. Figures P value and N number

Figure No.	Groups	*P* value	Significant	No. 1	No. 2	Methods
Fig 1d	Pre-wave vs. Ruffling	<0.0001	****	30	30	One way ANOVA
	Pre-wave vs. Increasing	0.3067	ns	30	30	One way ANOVA
	Pre-wave vs. Spreading	0.1136	ns	30	30	One way ANOVA
	Ruffling vs. Increasing	0.0093	**	30	30	One way ANOVA
	Ruffling vs. Spreading	<0.0001	****	30	30	One way ANOVA
	Increasing vs. Spreading	0.0006	***	30	30	One way ANOVA
Fig 1e	Pre-wave vs. Ruffling	<0.0001	****	110	51	One way ANOVA
	Pre-wave vs. Increasing	<0.0001	****	110	51	One way ANOVA
	Pre-wave vs. Spreading	<0.0001	****	110	59	One way ANOVA
	Ruffling vs. Increasing	<0.0001	****	51	51	One way ANOVA
	Ruffling vs. Spreading	0.0001	***	51	59	One way ANOVA
	Increasing vs. Spreading	0.9012	ns	51	59	One way ANOVA
Fig 1f	Pre-wave vs. Ruffling	<0.0001	****	20	20	One way ANOVA
	Pre-wave vs. Increasing	0.0007	***	20	20	One way ANOVA
	Pre-wave vs. Spreading	0.0476	*	20	20	One way ANOVA
	Ruffling vs. Increasing	<0.0001	****	20	20	One way ANOVA
	Ruffling vs. Spreading	<0.0001	****	20	20	One way ANOVA
	Increasing vs. Spreading	0.4987	ns	20	20	One way ANOVA
Fig 1h	Area					
	Pre-wave vs. Ruffling	0.2708	ns	20	20	One way ANOVA
	Pre-wave vs. Increasing	<0.0001	****	20	20	One way ANOVA
	Ruffling vs. Increasing	<0.0001	****	20	20	One way ANOVA
	Perimeter					
	Pre-wave vs. Ruffling	<0.0001	****	20	20	One way ANOVA
	Pre-wave vs. Increasing	<0.0001	****	20	20	One way ANOVA
	Ruffling vs. Increasing	0.1224	ns	20	20	One way ANOVA
Fig 1i	Pre-wave vs. Ruffling	0.9148	ns	41	50	One way ANOVA
	Pre-wave vs. Increasing	<0.0001	****	41	40	One way ANOVA
	Pre-wave vs. Spreading	<0.0001	****	41	70	One way ANOVA
	Ruffling vs. Increasing	<0.0001	****	50	40	One way ANOVA
	Ruffling vs. Spreading	<0.0001	****	50	70	One way ANOVA
	Increasing vs. Spreading	<0.0001	****	40	70	One way ANOVA
Fig 1j	Pre-wave vs. Ruffling	0.1703	ns	20	20	One way ANOVA
	Pre-wave vs. Increasing	<0.0001	****	20	20	One way ANOVA
	Pre-wave vs. Spreading	<0.0001	****	20	26	One way ANOVA
	Ruffling vs. Increasing	<0.0001	****	20	20	One way ANOVA
	Ruffling vs. Spreading	<0.0001	****	20	26	One way ANOVA
	Increasing vs. Spreading	<0.0001	****	20	26	One way ANOVA
Fig 2c	Rac1 vs. Area	<0.0001	****	20	20	One way ANOVA
	Rac1 vs. F-actin	<0.0001	****	20	20	One way ANOVA

	Area vs. F-actin	<0.0001	****	20	20	One way ANOVA
Fig 2f	Area vs. Rho1	0.1048	ns	20	20	One way ANOVA
	Area vs. F-actin	<0.0001	****	20	20	One way ANOVA
	Rho1 vs. F-actin	<0.0001	****	20	20	One way ANOVA
Fig 2i	Area vs. Rho1	0.0023	**	20	20	One way ANOVA
	Area vs. F-actin	<0.0001	****	20	20	One way ANOVA
	Rho1 vs. F-actin	<0.0001	****	20	20	One way ANOVA
Fig 2l	Area vs. F-actin	<0.0001	****	10	10	One way ANOVA
	Area vs. Talin	<0.0001	****	10	10	One way ANOVA
	F-actin vs. Talin	<0.0001	****	10	10	One way ANOVA
Fig 2o	Talin vs. F-actin	<0.0001	****	15	15	t test
Fig 3g	WT vs. Rac1 DN	<0.0001	****	20	20	One way ANOVA
	WT vs. Rho1 DN	0.9992	ns	20	20	One way ANOVA
	RacDN vs. Rho1 DN	<0.0001	****	20	20	One way ANOVA
Fig 3h	WT vs. Rac1 DN	0.034	*	10	10	One way ANOVA
	WT vs. Rho1 DN	0.6961	ns	10	10	One way ANOVA
	Rac1 DN vs. Rho1 DN	0.1763	ns	10	10	One way ANOVA
Fig 3i	WT vs. Rac1 DN	<0.0001	****	30	25	One way ANOVA
	WT vs. Rho1 DN	<0.0001	****	30	30	One way ANOVA
	Rac1 DN vs. Rho1 DN	0.002	**	25	30	One way ANOVA
Fig 3j	WT vs. Rac1 DN	<0.0001	****	15	15	One way ANOVA
	WT vs. Rho1 DN	<0.0001	****	15	15	One way ANOVA
	Rac1 DN vs. Rho1 DN	0.3765	ns	15	15	One way ANOVA
Fig 3k	WT vs. Rac1 DN	<0.0001	****	23	15	One way ANOVA
	WT vs. Rho1 DN	<0.0001	****	23	15	One way ANOVA
	Rac1 DN vs. Rho1 DN	0.3822	ns	15	15	One way ANOVA
Fig 3l	WT vs. Pax OE	0.0005	***	20	20	One way ANOVA
	WT vs. Talin RNAi	0.7913	ns	20	20	One way ANOVA
	Pax OE vs. Talin RNAi	0.0039	**	20	20	One way ANOVA
Fig 3m	WT vs. Pax OE	<0.0001	****	30	32	One way ANOVA
	WT vs. Talin RNAi	<0.0001	****	30	40	One way ANOVA
	Pax OE vs. Talin RNAi	<0.0001	****	32	40	One way ANOVA
Fig 3n	WT vs. Pax OE	<0.0001	****	15	10	One way ANOVA
	WT vs. Talin RNAi	<0.0001	****	15	10	One way ANOVA
	Pax OE vs. Talin RNAi	<0.0001	****	10	10	One way ANOVA

Fig 3o	WT vs. Pax OE	0.7287	ns	10	10	One way ANOVA
	WT vs. Talin RNAi	0.0058	**	10	10	One way ANOVA
	Pax OE vs. Talin RNAi	0.0353	*	10	10	One way ANOVA
Fig 4b	Pre-wave vs. Ruffling	0.7293	ns	41	50	One way ANOVA
	Pre-wave vs. Increasing	<0.0001	****	41	40	One way ANOVA
	Pre-wave vs. Spreading	<0.0001	****	41	70	One way ANOVA
	Ruffling vs. Increasing	<0.0001	****	50	40	One way ANOVA
	Ruffling vs. Spreading	<0.0001	****	50	70	One way ANOVA
	Increasing vs. Spreading	<0.0001	****	40	70	One way ANOVA
Fig 4f	FCs					
	Pre-wave vs. Ruffling	0.3554	ns	45	38	One way ANOVA
	Pre-wave vs. Spreading	<0.0001	****	45	51	One way ANOVA
	Ruffling vs. Spreading	<0.0001	****	38	51	One way ANOVA
	Ocytes					
	Pre-wave vs. Ruffling	0.9182	ns	11	15	One way ANOVA
	Pre-wave vs. Spreading	<0.0001	****	11	20	One way ANOVA
	Ruffling vs. Spreading	<0.0001	****	15	20	One way ANOVA
Fig 4h	WT vs. Rac1 DN	<0.0001	****	10	10	One way ANOVA
	WT vs. Rho1 DN	>0.9999	ns	10	10	One way ANOVA
	Rac1 DN vs. Rho1 DN	<0.0001	****	10	10	One way ANOVA
Fig 4i	WT vs. Rac1 DN	0.0004	***	30	25	One way ANOVA
	WT vs. Rho1 DN	<0.0001	****	30	30	One way ANOVA
	Rac1 DN vs. Rho1 DN	0.0089	**	25	30	One way ANOVA
Fig 4k	Left					
	WT vs. Rac1 DN	<0.0001	****	20	44	One way ANOVA
	WT vs. Rho1 DN	<0.0001	****	20	35	One way ANOVA
	Rac1 DN vs. Rho1 DN	0.6196	ns	44	35	One way ANOVA
	Right					
	WT vs. Pax OE	0.8444	ns	14	17	One way ANOVA
	WT vs. Talin RNAi	<0.0001	****	14	26	One way ANOVA
	Pax OE vs. Talin RNAi	<0.0001	****	17	26	One way ANOVA
Fig 5b	WT vs. Singed mutant	<0.0001	****	22	22	One way ANOVA
	WT vs. Singed RNAi	<0.0001	****	22	21	One way ANOVA
	Singed mutant vs. Singed RNAi	0.0068	**	22	21	One way ANOVA
Fig 5c	WT vs. Singed mutant	<0.0001	****	22	22	One way ANOVA
	WT vs. Singed RNAi	<0.0001	****	22	21	One way ANOVA
	Singed mutant vs. Singed RNAi	0.2741	ns	22	21	One way ANOVA
Fig 5l	WT vs. Singed mutant	<0.0001	****	20	25	One way ANOVA
	WT vs. Singed RNAi	<0.0001	****	20	20	One way ANOVA
	Singed mutant vs. Singed RNAi	0.728	ns	25	20	One way ANOVA

Fig 5m	WT vs. Singed mutant	<0.0001	****	20	25	One way ANOVA
	WT vs. Singed RNAi	<0.0001	****	20	20	One way ANOVA
	Singed mutant vs. Singed RNAi	0.8915	ns	25	20	One way ANOVA
Fig 6b	Pre-wave vs. Spreading	<0.0001	****	24	18	t test
Supplementary Figure 1d	Ruffling					
	Myosin vs. F-actin	0.3259	ns	15	15	t test
	Increasing					
	Myosin vs. F-actin	0.1534	ns	15	15	t test
	Spreading					
	Myosin vs. F-actin	All the values are zero	ns	15	15	t test
Supplementary Figure 1f	Pre-wave vs. Ruffling	<0.0001	****	68	53	One way ANOVA
	Pre-wave vs. Increasing	0.9858	ns	68	50	One way ANOVA
	Pre-wave vs. Spreading	0.0684	ns	68	50	One way ANOVA
	Ruffling vs. Increasing	<0.0001	****	53	50	One way ANOVA
	Ruffling vs. Spreading	<0.0001	****	53	50	One way ANOVA
	Increasing vs. Spreading	0.201	ns	50	50	One way ANOVA
Supplementary Figure 2f	Basal vs. Apical	0.7757	ns	10	10	One way ANOVA
	Basal vs. Sub basal	>0.9999	ns	10	10	One way ANOVA
	Apical vs. Sub basal	0.7757	ns	10	10	One way ANOVA
Supplementary Figure 2g		All the values are zero	ns	10	10	One way ANOVA
Supplementary Figure 3b	Center distance ratio					
	Ruffling vs. Spreading	0.0207	*	20	17	t test
	Area ratio					
	Ruffling vs. Spreading	<0.0001	****	20	17	t test
Supplementary Figure 3e	Area vs. Myosin delay	0.0014	**	15	15	One way ANOVA
	Area vs. Myosin Latency	<0.0001	****	15	15	One way ANOVA
	Myosin delay vs. Myosin Latency	<0.0001	****	15	15	One way ANOVA
Supplementary Figure 3h	Area vs. Myosin delay	0.0001	***	15	15	One way ANOVA
	Area vs. Myosin Latency	<0.0001	****	15	15	One way ANOVA
	Myosin delay vs. Myosin Latency	<0.0001	****	15	15	One way ANOVA
Supplementary Figure 3p	Area decrease vs. Myosin increase	<0.0001	****	10	10	t test

Supplementary Figure 4b	Pre-wave vs. Ruffling	0.0002	***	20	20	One way ANOVA
	Pre-wave vs. Increasing	0.9048	ns	20	20	One way ANOVA
	Pre-wave vs. Spreading	0.0002	***	20	20	One way ANOVA
	Ruffling vs. Increasing	0.0017	**	20	20	One way ANOVA
	Ruffling vs. Spreading	>0.9999	ns	20	20	One way ANOVA
	Increasing vs. Spreading	0.0017	**	20	20	One way ANOVA
Supplementary Figure 4c	Pre-wave vs. Ruffling	0.0003	***	20	20	One way ANOVA
	Pre-wave vs. Increasing	<0.0001	****	20	20	One way ANOVA
	Pre-wave vs. Spreading	<0.0001	****	20	20	One way ANOVA
	Ruffling vs. Increasing	0.0006	***	20	20	One way ANOVA
	Ruffling vs. Spreading	0.0294	*	20	20	One way ANOVA
	Increasing vs. Spreading	0.5943	ns	20	20	One way ANOVA
Supplementary Figure 4f	Pre-wave vs. Ruffling	0.0006	***	20	20	One way ANOVA
	Pre-wave vs. Increasing	0.4500	ns	20	21	One way ANOVA
	Pre-wave vs. Spreading	0.0002	***	20	20	One way ANOVA
	Ruffling vs. Increasing	0.0459	*	20	21	One way ANOVA
	Ruffling vs. Spreading	<0.0001	****	20	20	One way ANOVA
	Increasing vs. Spreading	<0.0001	****	21	20	One way ANOVA
Supplementary Figure 4i	Center distance ratio					
	Ruffling vs. Spreading	0.004	***	15	13	t test
	Area ratio					
	Ruffling vs. Spreading	<0.0001	****	15	13	t test
Supplementary Figure 4l	Pre-wave vs. Ruffling	0.0002	***	20	20	One way ANOVA
	Pre-wave vs. Increasing	0.0111	*	20	20	One way ANOVA
	Pre-wave vs. Spreading	>0.9999	ns	20	20	One way ANOVA
	Ruffling vs. Increasing	0.5957	ns	20	20	One way ANOVA
	Ruffling vs. Spreading	0.0002	***	20	20	One way ANOVA
	Increasing vs. Spreading	0.0112	*	20	20	One way ANOVA
Supplementary Figure 4m	Ruffling					
	About to Wave vs. Wave	0.0243	*	10	10	t test
	Spreading					
	About to Wave vs. Wave	0.002	***	10	10	t test
Supplementary Figure 5b	Low Rac vs. High Rac	<0.0001	****	11	9	t test
Supplementary Figure 5c	Ruffling area vs. No ruffling area	0.0003	***	12	20	t test
Supplementary Figure 5f	F-actin vs. Talin	<0.0001	****	15	15	t test

Supplementary Figure 6d	PA-RacQ61L					
	Without Laser 30 min vs. Without Laser 0 min	0.882	ns	19	19	t test
	With Laser 30 min vs. With Laser 0 min	0.001	**	18	18	t test
	PA-RacT17N					
	Without Laser 25 min vs. Without Laser 0 min	0.0037	**	15	15	t test
	With Laser 25 min vs. With Laser 0 min	0.0005	***	16	16	t test
Supplementary Figure 6g	WT vs. OptoRhoGEF	<0.0001	****	20	20	One way ANOVA
	WT vs. OptoRhoDN	<0.0001	****	20	20	One way ANOVA
	OptoRhoGEF vs. OptoRhoDN	<0.0001	****	20	20	One way ANOVA
Supplementary Figure 6l	WT vs. Rock RNAi	0.8367	ns	20	20	One way ANOVA
	WT vs. Sqh RNAi	0.8337	ns	20	20	One way ANOVA
	WT vs. Scar RNAi	<0.0001	****	20	20	One way ANOVA
	WTt vs. Arp3 RNAi	<0.0001	****	20	20	One way ANOVA
	WT vs. Abi RNAi	<0.0001	****	20	20	One way ANOVA
	Rock RNAi vs. Sqh RNAi	>0.9999	ns	20	20	One way ANOVA
	Rock RNAi vs. Scar RNAi	<0.0001	****	20	20	One way ANOVA
	Rock RNAi vs. Arp3 RNAi	<0.0001	****	20	20	One way ANOVA
	Rock RNAi vs. Abi RNAi	<0.0001	****	20	20	One way ANOVA
	Sqh RNAi vs. Scar RNAi	<0.0001	****	20	20	One way ANOVA
	Sqh RNAi vs. Arp3 RNAi	<0.0001	****	20	20	One way ANOVA
	Sqh RNAi vs. Abi RNAi	<0.0001	****	20	20	One way ANOVA
	Scar RNAi vs. Arp3 RNAi	0.8107	ns	20	20	One way ANOVA
	Scar RNAi vs. Abi RNAi	0.9998	ns	20	20	One way ANOVA
	Arp3 RNAi vs. Abi RNAi	0.6459	ns	20	20	One way ANOVA
Supplementary Figure 6m	WT vs. Rock RNAi	<0.0001	****	20	20	One way ANOVA
	WT vs. Scar RNAi	<0.0001	****	20	20	One way ANOVA
	WTt vs. Arp3 RNAi	<0.0001	****	20	20	One way ANOVA
	WT vs. Abi RNAi	<0.0001	****	20	20	One way ANOVA
	WT vs. Sqh RNAi	<0.0001	****	20	20	One way ANOVA
	Rock RNAi vs. Scar RNAi	0.9972	ns	20	20	One way ANOVA
	Rock RNAi vs. Arp3 RNAi	0.0552	ns	20	20	One way ANOVA
	Rock RNAi vs. Abi RNAi	0.1355	ns	20	20	One way ANOVA
	Rock RNAi vs. Sqh RNAi	0.8971	ns	20	20	One way ANOVA
	Sqh RNAi vs. Scar RNAi	0.0146	*	20	20	One way ANOVA
	Sqh RNAi vs. Arp3 RNAi	0.0428	*	20	20	One way ANOVA
	Sqh RNAi vs. Abi RNAi	0.6501	ns	20	20	One way ANOVA
	Scar RNAi vs. Arp3 RNAi	0.9991	ns	20	20	One way ANOVA
	Scar RNAi vs. Abi RNAi	0.4672	ns	20	20	One way ANOVA

	Arp3 RNAi vs. Abi RNAi	0.7065	ns	20	20	One way ANOVA
Supplementary Figure 7b	Pre-Wave					
	WT vs. Pax OE	<0.0001	****	11	20	One way ANOVA
	WT vs. RhoDN	0.0018	**	11	16	One way ANOVA
	WT vs. Talin RNAi	0.0076	**	11	20	One way ANOVA
	Pax OE vs. RhoDN	<0.0001	****	20	16	One way ANOVA
	Pax OE vs. Talin RNAi	<0.0001	****	20	20	One way ANOVA
	RhoDN vs. Talin RNAi	0.9661	ns	16	20	One way ANOVA
	Ruffling					
	WT vs. Pax OE	0.0002	***	11	20	One way ANOVA
	WT vs. RhoDN	0.8483	ns	11	16	One way ANOVA
	WT vs. Talin RNAi	0.6476	ns	11	20	One way ANOVA
	Pax OE vs. RhoDN	<0.0001	****	20	16	One way ANOVA
	Pax OE vs. Talin RNAi	0.001	**	20	20	One way ANOVA
	RhoDN vs. Talin RNAi	0.119	ns	16	20	One way ANOVA
	Spreading					
	WT vs. Pax OE	<0.0001	****	11	20	One way ANOVA
	WT vs. RhoDN	0.0021	**	11	16	One way ANOVA
	WT vs. Talin RNAi	0.7122	ns	11	20	One way ANOVA
	Pax OE vs. RhoDN	<0.0001	****	20	16	One way ANOVA
	Pax OE vs. Talin RNAi	<0.0001	****	20	20	One way ANOVA
	RhoDN vs. Talin RNAi	0.0455	*	16	20	One way ANOVA
Supplementary Figure 7e	WT vs. Pax OE	0.0497	*	10	10	One way ANOVA
	WT vs. Talin RNAi	0.0015	**	10	10	One way ANOVA
	WT vs. RhoDN	0.0045	**	10	10	One way ANOVA
	Pax OE vs. Talin RNAi	<0.0001	****	10	10	One way ANOVA
	Pax OE vs. RhoDN	<0.0001	****	10	10	One way ANOVA
	Talin RNAi vs. RhoDN	0.9773	ns	10	10	One way ANOVA
Supplementary Figure 7g	Convex area vs. Concave area	<0.0001	****	22	22	t test
Supplementary Figure 8a	Pre-wave vs. Ruffling	0.4473	ns	41	50	One way ANOVA
	Pre-wave vs. Increasing	0.2375	ns	41	40	One way ANOVA
	Pre-wave vs. Spreading	0.002	**	41	70	One way ANOVA
	Ruffling vs. Increasing	0.0038	**	50	40	One way ANOVA
	Ruffling vs. Spreading	<0.0001	****	50	70	One way ANOVA
	Increasing vs. Spreading	0.4412	ns	40	70	One way ANOVA
Supplementary Figure 8b	Pre-wave vs. Ruffling	0.0054	**	41	50	One way ANOVA
	Pre-wave vs. Increasing	<0.0001	****	41	40	One way ANOVA
	Pre-wave vs. Spreading	<0.0001	****	41	70	One way ANOVA
	Ruffling vs. Increasing	<0.0001	****	50	40	One way ANOVA
	Ruffling vs. Spreading	<0.0001	****	50	70	One way ANOVA
	Increasing vs. Spreading	<0.0001	****	40	70	One way ANOVA

Supplementary Figure 8f	FCs					
	Pre-wave vs. Ruffling	0.6357	ns	45	38	One way ANOVA
	Pre-wave vs. Spreading	<0.0001	****	45	51	One way ANOVA
	Ruffling vs. Spreading	<0.0001	****	38	51	One way ANOVA
	Ocytes					
	Pre-wave vs. Ruffling	0.0217	*	11	15	One way ANOVA
	Pre-wave vs. Spreading	<0.0001	****	11	20	One way ANOVA
	Ruffling vs. Spreading	<0.0001	****	15	20	One way ANOVA
Supplementary Figure 8g	FCs					
	Pre-wave vs. Ruffling	0.3324	ns	45	38	One way ANOVA
	Pre-wave vs. Spreading	<0.0001	****	45	51	One way ANOVA
	Ruffling vs. Spreading	<0.0001	****	38	51	One way ANOVA
	Ocytes	0.2836	ns			
	Pre-wave vs. Ruffling	<0.0001	****	11	15	One way ANOVA
	Pre-wave vs. Spreading	<0.0001	****	11	20	One way ANOVA
	Ruffling vs. Spreading			15	20	One way ANOVA
Supplementary Figure 8j	WT vs. Rac1 DN	0.0332	*	20	44	One way ANOVA
	WT vs. Rho1 DN	<0.0001	****	20	35	One way ANOVA
	Rac1 DN vs. Rho1 DN	<0.0001	****	44	35	One way ANOVA
	WT vs. Pax OE	0.0057	**	14	17	One way ANOVA
	WT vs. Talin RNAi	<0.0001	****	14	26	One way ANOVA
	Pax OE vs. Talin RNAi	<0.0001	****	17	26	One way ANOVA
Supplementary Figure 10b	Pre-wave vs. Ruffling	<0.0001	****	127	127	One way ANOVA
	Pre-wave vs. Increasing	<0.0001	****	127	127	One way ANOVA
	Pre-wave vs. Spreading	<0.0001	****	127	127	One way ANOVA
	Ruffling vs. Increasing	<0.0001	****	127	127	One way ANOVA
	Ruffling vs. Spreading	<0.0001	****	127	127	One way ANOVA
	Increasing vs. Spreading	<0.0001	****	127	127	One way ANOVA
Supplementary Figure 10c	Pre-wave vs. Ruffling	0.1778	ns	127	127	One way ANOVA
	Pre-wave vs. Increasing	<0.0001	****	127	127	One way ANOVA
	Pre-wave vs. Spreading	<0.0001	****	127	127	One way ANOVA
	Ruffling vs. Increasing	<0.0001	****	127	127	One way ANOVA
	Ruffling vs. Spreading	<0.0001	****	127	127	One way ANOVA
	Increasing vs. Spreading	<0.0001	****	127	127	One way ANOVA
Supplementary Figure 10d	Pre-wave vs. Ruffling	0.9782	ns	127	127	One way ANOVA
	Pre-wave vs. Increasing	<0.0001	****	127	127	One way ANOVA
	Pre-wave vs. Spreading	<0.0001	****	127	127	One way ANOVA
	Ruffling vs. Increasing	<0.0001	****	127	127	One way ANOVA
	Ruffling vs. Spreading	<0.0001	****	127	127	One way ANOVA

	Increasing vs. Spreading	<0.0001	****	127	127	One way ANOVA
Supplementary Figure 10e	Pre-wave vs. Ruffling	0.8519	ns	127	127	One way ANOVA
	Pre-wave vs. Increasing	<0.0001	****	127	127	One way ANOVA
	Pre-wave vs. Spreading	<0.0001	****	127	127	One way ANOVA
	Ruffling vs. Increasing	<0.0001	****	127	127	One way ANOVA
	Ruffling vs. Spreading	<0.0001	****	127	127	One way ANOVA
	Increasing vs. Spreading	0.3915	ns	127	127	One way ANOVA
Supplementary Figure 10f	Pre-wave vs. Ruffling	0.9976	ns	18	18	One way ANOVA
	Pre-wave vs. Increasing	0.6449	ns	18	18	One way ANOVA
	Pre-wave vs. Spreading	0.0119	*	18	18	One way ANOVA
	Ruffling vs. Increasing	0.7583	ns	18	18	One way ANOVA
	Ruffling vs. Spreading	0.0203	*	18	18	One way ANOVA
	Increasing vs. Spreading	0.1994	ns	18	18	One way ANOVA
Supplementary Figure 11c	WT vs. Rho1 DN	0.9999	ns	127	127	One way ANOVA
	WT vs. Rac1 DN Weak	0.9977	ns	127	127	One way ANOVA
	WT vs. Dumping inhibition	0.9989	ns	127	127	One way ANOVA
	WT vs. Pax OE	>0.9999	ns	127	127	One way ANOVA
	WT vs. Talin RNAi	0.9299	ns	127	127	One way ANOVA
	Rho1 DN vs. Rac1 DN	>0.9999	ns	127	127	One way ANOVA
	Rho1 DN vs. Dumping inhibition	0.9885	ns	127	127	One way ANOVA
	Rho1 DN vs. Pax OE	0.998	ns	127	127	One way ANOVA
	Rho1 DN vs. Talin RNAi	0.8307	ns	127	127	One way ANOVA
	Rac1 DN Weak vs. Dumping inhibition	0.962	ns	127	127	One way ANOVA
	Rac1 DN Weak vs. Pax OE	0.9887	ns	127	127	One way ANOVA
	Rac1 DN Weak vs. Talin RNAi	0.7212	ns	127	127	One way ANOVA
	Dumping inhibition vs. Pax OE	>0.9999	ns	127	127	One way ANOVA
	Dumping inhibition vs. Talin RNAi	0.9923	ns	127	127	One way ANOVA
	Pax OE vs. Talin RNAi	0.9711	ns	127	127	One way ANOVA
Supplementary Figure 11e	WT vs. Rho1 DN	0.0016	**	127	127	One way ANOVA
	WT vs. Rac1 DN Weak	<0.0001	****	127	127	One way ANOVA
	WT vs. Dumping inhibition	0.8827	ns	127	127	One way ANOVA
	WT vs. Pax OE	0.0003	***	127	127	One way ANOVA
	WT vs. Talin RNAi	0.168	ns	127	127	One way ANOVA
	Rho1 DN vs. Rac1 DN Weak	<0.0001	****	127	127	One way ANOVA
	Rho1 DN vs. Dumping inhibition	0.1413	ns	127	127	One way ANOVA
	Rho1 DN vs. Pax OE	<0.0001	****	127	127	One way ANOVA
	Rho1 DN vs. Talin RNAi	0.6244	ns	127	127	One way ANOVA
	Rac1 DN Weak vs. Dumping inhibition	<0.0001	****	127	127	One way ANOVA

	Rac1 DN Weak vs. Pax OE	<0.0001	****	127	127	One way ANOVA
	Rac1 DN Weak vs. Talin RNAi	<0.0001	****	127	127	One way ANOVA
	Dumping inhibition vs. Pax OE	<0.0001	****	127	127	One way ANOVA
	Dumping inhibition vs. Talin RNAi	0.8779	ns	127	127	One way ANOVA
	Pax OE vs. Talin RNAi	<0.0001	****	127	127	One way ANOVA
Supplementary Figure 11f	WT vs. Rho1 DN	<0.0001	****	36	36	One way ANOVA
	WT vs. Rac1 DN Weak	<0.0001	****	36	36	One way ANOVA
	WT vs. Dumping inhibition	<0.0001	****	36	36	One way ANOVA
	WT vs. Pax OE	0.0002	***	36	36	One way ANOVA
	WT vs. Talin RNAi	0.0033	**	36	36	One way ANOVA
	Rho1 DN vs. Rac1 DN Weak	<0.0001	****	36	36	One way ANOVA
	Rho1 DN vs. Dumping inhibition	<0.0001	****	36	36	One way ANOVA
	Rho1 DN vs. Pax OE	<0.0001	****	36	36	One way ANOVA
	Rho1 DN vs. Talin RNAi	<0.0001	****	36	36	One way ANOVA
	Rac1 DN Weak vs. Dumping inhibition	<0.0001	****	36	36	One way ANOVA
	Rac1 DN Weak vs. Pax OE	<0.0001	****	36	36	One way ANOVA
	Rac1 DN Weak vs. Talin RNAi	<0.0001	****	36	36	One way ANOVA
	Dumping inhibition vs. Pax OE	<0.0001	****	36	36	One way ANOVA
	Dumping inhibition vs. Talin RNAi	<0.0001	****	36	36	One way ANOVA
	Pax OE vs. Talin RNAi	<0.0001	****	36	36	One way ANOVA
Supplementary Figure 11g	WT vs. Rho1 DN	<0.0001	****	127	127	One way ANOVA
	WT vs. Rac1 DN Weak	<0.0001	****	127	127	One way ANOVA
	WT vs. Dumping inhibition	<0.0001	****	127	127	One way ANOVA
	WT vs. Pax OE	<0.0001	****	127	127	One way ANOVA
	WT vs. Talin RNAi	<0.0001	****	127	127	One way ANOVA
	Rho1 DN vs. Rac1 DN Weak	<0.0001	****	127	127	One way ANOVA
	Rho1 DN vs. Dumping inhibition	<0.0001	****	127	127	One way ANOVA
	Rho1 DN vs. Pax OE	<0.0001	****	127	127	One way ANOVA
	Rho1 DN vs. Talin RNAi	0.0022	**	127	127	One way ANOVA
	Rac1 DN vs. Dumping inhibition	0.0007	***	127	127	One way ANOVA
	Rac1 DN Weak vs. Pax OE	<0.0001	****	127	127	One way ANOVA
	Rac1 DN Weak vs. Talin RNAi	<0.0001	****	127	127	One way ANOVA
	Dumping inhibition vs. Pax OE	<0.0001	****	127	127	One way ANOVA
	Dumping inhibition vs. Talin RNAi	<0.0001	****	127	127	One way ANOVA
	Pax OE vs. Talin RNAi	<0.0001	****	127	127	One way ANOVA

Supplementary Figure 12d	WT vs. Rho1 DN	0.0025	**	127	127	One way ANOVA
	WT vs. Rac1 DN Weak	<0.0001	****	127	127	One way ANOVA
	WT vs. Rac1 DN Strong	<0.0001	****	127	127	One way ANOVA
	WT vs. Dumping inhibition	0.8788	ns	127	127	One way ANOVA
	WT vs. Pax OE	<0.0001	****	127	127	One way ANOVA
	WT vs. Talin RNAi	0.1865	ns	127	127	One way ANOVA
	Rho1 DN vs. Rac1 DN Weak	<0.0001	****	127	127	One way ANOVA
	Rho1 DN vs. Rac1 DN Strong	<0.0001	****	127	127	One way ANOVA
	Rho1 DN vs. Dumping inhibition	0.1273	ns	127	127	One way ANOVA
	Rho1 DN vs. Pax OE	<0.0001	****	127	127	One way ANOVA
	Rho1 DN vs. Talin RNAi	0.7985	ns	127	127	One way ANOVA
	Rac1 DN Weak vs. Rac1 DN Strong	<0.0001	****	127	127	One way ANOVA
	Rac1 DN Weak vs. Dumping inhibition	<0.0001	****	127	127	One way ANOVA
	Rac1 DN Weak vs. Pax OE	<0.0001	****	127	127	One way ANOVA
	Rac1 DN Weak vs. Talin RNAi	<0.0001	****	127	127	One way ANOVA
	Rac1 DN Strong vs. Dumping inhibition	<0.0001	****	127	127	One way ANOVA
	Rac1 DN Strong vs. Pax OE vs	<0.0001	****	127	127	One way ANOVA
	Rac1 DN Strong vs. Talin RNAi	<0.0001	****	127	127	One way ANOVA
	Dumping inhibition vs. Pax OE	<0.0001	****	127	127	One way ANOVA
	Dumping inhibition vs. Talin RNAi	0.8969	ns	127	127	One way ANOVA
	Pax OE vs. Talin RNAi	<0.0001	****	127	127	One way ANOVA
Supplementary Figure 12e	WT vs. Rho1 DN	0.9918	ns	127	127	One way ANOVA
	WT vs. Rac1 DN Weak	0.8716	ns	127	127	One way ANOVA
	WT vs. Rac1 DN Strong	0.083	ns	127	127	One way ANOVA
	WT vs. Dumping inhibition	0.9995	ns	127	127	One way ANOVA
	WT vs. Pax OE	>0.9999	ns	127	127	One way ANOVA
	WT vs. Talin RNAi	0.9999	ns	127	127	One way ANOVA
	Rho1 DN vs. Rac1 DN Weak	0.9982	ns	127	127	One way ANOVA
	Rho1 DN vs. Rac1 DN Strong	0.3793	ns	127	127	One way ANOVA
	Rho1 DN vs. Dumping inhibition	0.9127	ns	127	127	One way ANOVA
	Rho1 DN vs. Pax OE	0.9946	ns	127	127	One way ANOVA
	Rho1 DN vs. Talin RNAi	0.9434	ns	127	127	One way ANOVA
	Rac1 DN Weak vs. Rac1 DN Strong	0.7365	ns	127	127	One way ANOVA
	Rac1 DN Weak vs. Dumping inhibition	0.6217	ns	127	127	One way ANOVA
	Rac1 DN Weak vs. Pax OE	0.8925	ns	127	127	One way ANOVA
	Rac1 DN Weak vs. Talin RNAi	0.6901	ns	127	127	One way ANOVA
	Rac1 DN Strong vs. Dumping inhibition	0.0235	*	127	127	One way ANOVA

	Rac1 DN Strong vs. Pax OE vs	0.0948	ns	127	127	One way ANOVA
	Rac1 DN Strong vs. Talin RNAi	0.0324	*	127	127	One way ANOVA
	Dumping inhibition vs. Pax OE	0.999	ns	127	127	One way ANOVA
	Dumping inhibition vs. Talin RNAi	>0.9999	ns	127	127	One way ANOVA
	Pax OE vs. Talin RNAi	0.9998	ns	127	127	One way ANOVA
Supplementary Figure 12g	WT vs. Rho1 DN	<0.0001	****	36	36	One way ANOVA
	WT vs. Rac1 DN Weak	<0.0001	****	36	36	One way ANOVA
	WT vs. Rac1 DN Strong	<0.0001	****	36	36	One way ANOVA
	WT vs. Dumping inhibition	<0.0001	****	36	36	One way ANOVA
	WT vs. Pax OE	<0.0001	****	36	36	One way ANOVA
	WT vs. Talin RNAi	<0.0001	****	36	36	One way ANOVA
	Rho1 DN vs. Rac1 DN Weak	<0.0001	****	36	36	One way ANOVA
	Rho1 DN vs. Rac1 DN Strong	<0.0001	****	36	36	One way ANOVA
	Rho1 DN vs. Dumping inhibition	<0.0001	****	36	36	One way ANOVA
	Rho1 DN vs. Pax OE	<0.0001	****	36	36	One way ANOVA
	Rho1 DN vs. Talin RNAi	<0.0001	****	36	36	One way ANOVA
	Rac1 DN Weak vs. Rac1 DN Strong	<0.0001	****	36	36	One way ANOVA
	Rac1 DN Weak vs. Dumping inhibition	<0.0001	****	36	36	One way ANOVA
	Rac1 DN Weak vs. Pax OE	<0.0001	****	36	36	One way ANOVA
	Rac1 DN Weak vs. Talin RNAi	0.6161	ns	36	36	One way ANOVA
	Rac1 DN Strong vs. Dumping inhibition	<0.0001	****	36	36	One way ANOVA
	Rac1 DN Strong vs. Pax OE vs	<0.0001	****	36	36	One way ANOVA
	Rac1 DN Strong vs. Talin RNAi	<0.0001	****	36	36	One way ANOVA
	Dumping inhibition vs. Pax OE	<0.0001	****	36	36	One way ANOVA
	Dumping inhibition vs. Talin RNAi	<0.0001	****	36	36	One way ANOVA
	Pax OE vs. Talin RNAi	<0.0001	****	36	36	One way ANOVA
Supplementary Figure 12i	WT vs. Rho1 DN	<0.0001	****	127	127	One way ANOVA
	WT vs. Rac1 DN Weak	<0.0001	****	127	127	One way ANOVA
	WT vs. Rac1 DN Strong	<0.0001	****	127	127	One way ANOVA
	WT vs. Dumping inhibition	<0.0001	****	127	127	One way ANOVA
	WT vs. Pax OE	<0.0001	****	127	127	One way ANOVA
	WT vs. Talin RNAi	<0.0001	****	127	127	One way ANOVA
	Rho1 DN vs. Rac1 DN Weak	<0.0001	****	127	127	One way ANOVA
	Rho1 DN vs. Rac1 DN Strong	<0.0001	****	127	127	One way ANOVA
	Rho1 DN vs. Dumping inhibition	<0.0001	****	127	127	One way ANOVA
	Rho1 DN vs. Pax OE	<0.0001	****	127	127	One way ANOVA

	Rho1 DN vs. Talin RNAi	0.0001	***	127	127	One way ANOVA
	Rac1 DN Weak vs. Rac1 DN Strong	<0.0001	****	127	127	One way ANOVA
	Rac1 DN Weak vs. Dumping inhibition	0.0647	ns	127	127	One way ANOVA
	Rac1 DN Weak vs. Pax OE	<0.0001	****	127	127	One way ANOVA
	Rac1 DN Weak vs. Talin RNAi	>0.9999	ns	127	127	One way ANOVA
	Rac1 DN Strong vs. Dumping inhibition	<0.0001	****	127	127	One way ANOVA
	Rac1 DN Strong vs. Pax OE vs	<0.0001	****	127	127	One way ANOVA
	Rac1 DN strong vs. Talin RNAi	<0.0001	****	127	127	One way ANOVA
	Dumping inhibition vs. Pax OE	<0.0001	****	127	127	One way ANOVA
	Dumping inhibition vs. Talin RNAi	0.0301	*	127	127	One way ANOVA
	Pax OE vs. Talin RNAi	<0.0001	****	127	127	One way ANOVA

Supplementary References

1. Martin, A.C., Kaschube, M. & Wieschaus, E.F. Pulsed contractions of an actin-myosin network drive apical constriction. *Nature* **457**, 495-499 (2009).
2. Zhou, S. *et al.* Two Rac1 pools integrate the direction and coordination of collective cell migration. *Nat Commun* **13**, 6014 (2022).
3. Huang, J., Zhou, W., Dong, W., Watson, A.M. & Hong, Y. From the Cover: Directed, efficient, and versatile modifications of the Drosophila genome by genomic engineering. *Proc Natl Acad Sci U S A* **106**, 8284-8289 (2009).
4. Venken, K.J. *et al.* MiMIC: a highly versatile transposon insertion resource for engineering Drosophila melanogaster genes. *Nat Methods* **8**, 737-743 (2011).
5. Munjal, A., Philippe, J.M., Munro, E. & Lecuit, T. A self-organized biomechanical network drives shape changes during tissue morphogenesis. *Nature* **524**, 351-355 (2015).
6. Izquierdo, E., Quinkler, T. & De Renzis, S. Guided morphogenesis through optogenetic activation of Rho signalling during early Drosophila embryogenesis. *Nat Commun* **9**, 2366 (2018).
7. Guo, H., Swan, M. & He, B. Optogenetic inhibition of actomyosin reveals mechanical bistability of the mesoderm epithelium during Drosophila mesoderm invagination. *Elife* **11** (2022).
8. Qin, X. *et al.* Cell-matrix adhesion and cell-cell adhesion differentially control basal myosin oscillation and Drosophila egg chamber elongation. *Nat Commun* **8**, 14708 (2017).
