# Supplementary material for: Basal actomyosin pulses expand epithelium coordinating cell flattening and tissue elongation
Source: Nat Commun. 2024 Apr 8;15:3000. doi: 10.1038/s41467-024-47236-1 (PMC11001887; doi:10.1038/s41467-024-47236-1)
Supplement: Supplementary file 3 — Description of Additional Supplementary Files [file 41467_2024_47236_MOESM3_ESM.pdf]

Description of Additional Supplementary Files

Supplementary Movie 1. Follicle cell expansion behaviour during stage 10B to stage 12. Dorsal view time-lapse sequence of follicular epithelial cells expressing E-cadherin-GFP and Sqh-RFP in the WT egg chamber during the whole period of cell expansion waves. The unit of time is minutes. The beginning time points of different phases have been labelled. Scale bar is 10 μm.

Supplementary Movie 2. Different phases of follicle cell expansion behaviour. Time-lapse sequence of posterior follicular epithelial cells expressing E-cadherin-GFP and Sqh-RFP in the WT egg chambers during the 3 different phases. The unit of time is minutes. Scale bar is 5 μm.

Supplementary Movie 3. Focal adhesions and stress fibers during follicle cell expansion waves. Time-lapse sequence of follicular epithelial cells expressing Talin-GFP and Sqh-RFP in the WT egg chamber during the whole period of cell expansion waves. The beginning time points of different phases have been labelled. The unit of time is minutes. Scale bar is 10 μm.

Supplementary Movie 4. Follicle cell expansion waves in the WT background. Time-lapse sequence of follicular epithelial cells expressing E-cadherin-GFP and LifeAct-RFP in the WT epithelial background during the whole period of cell expansion waves. The beginning time points of different phases have been labelled. The unit of time is minutes. Scale bar is 10 μm.

Supplementary Movie 5. Follicle cell expansion waves in the Rac1 inhibition background. Time-lapse sequence of follicular epithelial cells expressing E-cadherin-GFP and LifeAct-RFP in the Rac1DN-inhibitory epithelial background during the whole period of cell expansion waves (defined by WT tissue). The unit of time is minutes. Scale bar is 10 μm.

Supplementary Movie 6. Follicle cell expansion waves in the Rho1 inhibition background. Time-lapse sequence of follicular epithelial cells expressing E-cadherin-GFP and LifeAct-RFP in the Rho1DN-inhibitory epithelial background during the whole period of cell expansion waves (defined by WT tissue). The unit of time is minutes. Scale bar is 10 μm.

Supplementary Movie 7. Follicle cell expansion waves in enhanced focal adhesion background. Time-lapse sequence of follicular epithelial cells expressing E-cadherin-GFP and LifeAct-RFP in the Paxillin-overexpression epithelial background during the whole period of cell expansion waves (defined by WT tissue). The unit of time is minutes. Scale bar is 10 μm.

Supplementary Movie 8. Follicle cell expansion waves in reduced focal adhesion background. Time-lapse sequence of follicular epithelial cells expressing E-cadherin-GFP and LifeAct-RFP in the Talin RNAi-expressing epithelial background during the whole period of cell expansion waves (defined by WT tissue). The unit of time is minutes. Scale bar is 10 μm.

Supplementary Movie 9. Super-solved dynamics of pulsatile basal actomyosin networks during the pre-wave phase. Time-lapse sequence of RIM super-solved images of basal LifeAct-GFP and Sqh-RFP signals in follicle cells during the pre-wave phase. The unit of time is second. Scale bar is 2 μm.

Supplementary Movie 10. Super-solved dynamics of pulsatile basal actomyosin networks during the spreading phase. Time-lapse sequence of RIM super-solved images of basal LifeAct-GFP and Sqh-RFP signals in follicle cells during the spreading phase. The unit of time is second. Scale bar is 2 μm.

Supplementary Movie 11. Super-solved dynamics of basal Myosin-II and focal adhesion signals during the pre-wave phase. Time-lapse sequence of RIM super-solved images of basal Talin-GFP and Sqh-RFP signals in follicle cells during the pre-wave phase. The unit of time is second. Scale bar is 2 μm.

Supplementary Movie 12. Super-solved dynamics of basal Myosin-II and focal adhesion signals during the spreading phase. Time-lapse sequence of RIM super-solved images of basal Talin-GFP and Sqh-RFP signals in follicle cells during the spreading phase. The unit of time is second. Scale bar is 2 μm.

Supplementary Movie 13. *In silico* simulation of follicle cell expansion waves in the WT egg chamber. Time-lapse sequence of follicular epithelial cell expansion waves at conformal projection view. Blue colour marks the cell boundary at basal domain, and red colour marks basal stress fibers during the increasing and spreading phases. The unit of time is seconds.

Supplementary Movie 14. *In silico* simulation of follicle cell expansion waves surrounding the oocyte at different genetic backgrounds. Time-lapse sequence of follicular epithelial cell expansion waves surrounding the oocyte at the indicated different genetic backgrounds. Green color marks the cell boundary at basal domain, and red color marks basal stress fibers during the spreading phase. The thickness of stress fibers represents the relative intensity of stress fibers: the stress fibers of Rho1 DN and Talin RNAi are reduced, while the stress fibers of Pax OE are increased. Dotted lines represent the final position of follicle cell expansion waves in the WT egg chambers. The unit of time is seconds.
