# Supplementary material for: Basal actomyosin pulses expand epithelium coordinating cell flattening and tissue elongation
Source: Nat Commun. 2024 Apr 8;15:3000. doi: 10.1038/s41467-024-47236-1 (PMC11001887; doi:10.1038/s41467-024-47236-1)
Supplement: Supplementary file 18 — Reporting Summary [file 41467_2024_47236_MOESM18_ESM.pdf]

nature portfolio

Corresponding author(s): Xiaobo Wang

Last updated by author(s): Feb 27, 2024

Reporting Summary

Nature Portfolio wishes to improve the reproducibility of the work that we publish. This form provides structure for consistency and transparency in reporting. For further information on Nature Portfolio policies, see our Editorial Policies and the Editorial Policy Checklist.

Statistics

For all statistical analyses, confirm that the following items are present in the figure legend, table legend, main text, or Methods section.

n/a	Confirmed	
☐	☒	The exact sample size (*n*) for each experimental group/condition, given as a discrete number and unit of measurement
☐	☒	A statement on whether measurements were taken from distinct samples or whether the same sample was measured repeatedly
☐	☒	The statistical test(s) used AND whether they are one- or two-sided<br>*Only common tests should be described solely by name; describe more complex techniques in the Methods section.*
☐	☒	A description of all covariates tested
☒	☐	A description of any assumptions or corrections, such as tests of normality and adjustment for multiple comparisons
☐	☒	A full description of the statistical parameters including central tendency (e.g. means) or other basic estimates (e.g. regression coefficient) AND variation (e.g. standard deviation) or associated estimates of uncertainty (e.g. confidence intervals)
☐	☒	For null hypothesis testing, the test statistic (e.g. *F*, *t*, *r*) with confidence intervals, effect sizes, degrees of freedom and *P* value noted<br>*Give P values as exact values whenever suitable.*
☒	☐	For Bayesian analysis, information on the choice of priors and Markov chain Monte Carlo settings
☒	☐	For hierarchical and complex designs, identification of the appropriate level for tests and full reporting of outcomes
☐	☒	Estimates of effect sizes (e.g. Cohen's *d*, Pearson's *r*), indicating how they were calculated

Our web collection on statistics for biologists contains articles on many of the points above.

Software and code

Policy information about availability of computer code

Data collection	Imaging data have been collected by Leica Metamorph software (version: Metamorph 7.8.13.0).
Data analysis	GraphPad Prism software (version: 8.0.2) has been used for box and whiskers plots. ImageJ software (version: 1.53c) has been used for quantifications. MATLAB software (version: R2020b) has been used for photo-bleaching correction, build heatmap of curvature, measure the cell morphology and perform the particle image velocimetry (PIV) analysis. The codes used for analyses of follicle cell features and signal intensity (named as: cell feature and intensity) can be found within the website: https://github.com/heishuiguo/cell-and-fiber-feature. The codes used for draw heatmap of curvature on the cell edge (named as: curvature) can be found within the website: https://github.com/heishuiguo/cell-and-fiber-feature. The codes used for analyses of relative angle of stress fibers (named as: polarity) can be found within the website: https://github.com/heishuiguo/cell-and-fiber-feature. The codes used for Isolation of D-V oriented fibrous signals (named as: isolation of D-V oriented fibrous signal) can be found within the website: https://github.com/heishuiguo/cell-and-fiber-feature. The custom codes for modelling and simulation are available from the corresponding authors upon reasonable request.

For manuscripts utilizing custom algorithms or software that are central to the research but not yet described in published literature, software must be made available to editors and reviewers. We strongly encourage code deposition in a community repository (e.g. GitHub). See the Nature Portfolio guidelines for submitting code & software for further information.

Data

Policy information about availability of data

All manuscripts must include a data availability statement. This statement should provide the following information, where applicable:

- Accession codes, unique identifiers, or web links for publicly available datasets
- A description of any restrictions on data availability
- For clinical datasets or third party data, please ensure that the statement adheres to our policy

The source data underlying Figs 1d-j, 2c,f,i,l,o, 3g-o, 4b,f,h,i,k, 5b,c,l,m, 6b, and Supplementary Figs 1d,f, 2f,g, 3b,e,h,p, 4b,c,f,i,l,m, 5f, 6d,g,j,l,m, 7b,e,g, 8a,b,f,g,j, 10b-f, 11c,e-g, 12d,e,g,i are provided as a Source Data file.
Complete data are available in the main article, supplementary materials, and source data files. Since all the raw confocal imaging data supporting the findings of this study runs more than four terabytes and in multiple files, we have not submitted it to the public repository but preserved in our NAS drive and are freely available from the corresponding author (Contact Address: xiaobo.wang@univ-tlse3.fr). Representative images are in the main or supplementary figures. Source data are provided with this paper.

Research involving human participants, their data, or biological material

Policy information about studies with human participants or human data. See also policy information about sex, gender (identity/presentation), and sexual orientation and race, ethnicity and racism.

Reporting on sex and gender	No human participants or human data were used
Reporting on race, ethnicity, or other socially relevant groupings	No human participants or human data were used
Population characteristics	No human participants or human data were used
Recruitment	No human participants or human data were used
Ethics oversight	No human participants or human data were used

Note that full information on the approval of the study protocol must also be provided in the manuscript.

Field-specific reporting

Please select the one below that is the best fit for your research. If you are not sure, read the appropriate sections before making your selection.

[x] Life sciences [ ] Behavioural & social sciences [ ] Ecological, evolutionary & environmental sciences

For a reference copy of the document with all sections, see nature.com/documents/nr-reporting-summary-flat.pdf

Life sciences study design

All studies must disclose on these points even when the disclosure is negative.

Sample size	The experiments were performed, in general, on the 50-200 follicle cells and from 10-50 independent tissue samples for most studies, and on at least 8 independent samples for optogenetics. The exact number of analyzed samples is specified for each experiment in the corresponding figure and/or figure legends.
Data exclusions	No data were excluded from the analysis.
Replication	The experiments were replicated or performed independently at least 3 times, and the exact number of independent experiment is listed in the corresponding figures or legends (also included in Supplementary Note 1).
Randomization	Sample allocation was random.
Blinding	We were blinded to group allocation during data collection and analysis.

Reporting for specific materials, systems and methods

We require information from authors about some types of materials, experimental systems and methods used in many studies. Here, indicate whether each material, system or method listed is relevant to your study. If you are not sure if a list item applies to your research, read the appropriate section before selecting a response.

Materials & experimental systems

n/a	Involved in the study
x	Antibodies
x	Eukaryotic cell lines
x	Palaeontology and archaeology
	x Animals and other organisms
x	Clinical data
x	Dual use research of concern
x	Plants

Methods

n/a	Involved in the study
x	ChIP-seq
x	Flow cytometry
x	MRI-based neuroimaging

Animals and other research organisms

Policy information about studies involving animals; ARRIVE guidelines recommended for reporting animal research, and Sex and Gender in Research

Laboratory animals	Drosophila melanogaster, both male and female, 3-5 days after adult flies are born. The following fly stocks were used (information is listed in Supplementary Table 1): Sqh::RLCmyosinII–mCherry (from Eric E. Wieschaus), slbo::LifeAct–GFP (from our previous study), slbo::LifeAct–RFP (from our previous study), E-cadherin-GFP (from Hong Yong), Talin-GFP (from Yohanns Bellaiche), UASt-PA-RacCA (from our previous study), UASt-PA-RacDN (from our previous study), UASp-CIBN-CAAX/UASp-Cry2-RhoGEF (Opto-RhoGEF tool from Stefano De Renzis), UASp-CIBN-CAAX/UASp-Cry2-Rho1DN (Opto-Rho1DN tool from Bing He), Ubi::AniRBD-GFP (from Thomas Lecuit), Singed2/FM7 (from Zennifer Zanet), UAS-Paxillin (from Christos G. Zervas), UAS-Rac1DN (BL6292), UAS-Rho1DN (BL7327), UAS-ROCKRNAi (BL34324), UAS-SqhRNAi (BL32439), UAS-ScarRNAi (BL51803), UAS-AbiRNAi (BL51455) , UAS-Arp3RNAi (BL32921), UAS-TalinRNAi (BL33913), UAS-SingedRNAi (BL42615), and Sqh::PAK3-RBD-GFP (BL52303/BL52304).
Wild animals	No wild animals were used in the study.
Reporting on sex	Sex was not considered in this study design, method used for assigning sex
Field-collected samples	No field collected samples were used in the study.
Ethics oversight	Ethical approval was not required for this study

Note that full information on the approval of the study protocol must also be provided in the manuscript.

Plants

Seed stocks	No plants was used
Novel plant genotypes	No plants was used
Authentication	No plants was used
